# Physiological and image-based phenotyping assessment of waterlogging responses of three kiwifruit rootstocks and grafting combinations

**DOI:** 10.3389/fpls.2025.1499432

**Published:** 2025-02-05

**Authors:** Maria Calabritto, Alba N. Mininni, Roberto Di Biase, Angelo Petrozza, Stephan Summerer, Francesco Cellini, Bartolomeo Dichio

**Affiliations:** ^1^ Department of Agricultural, Forest, Food, and Environmental Sciences (DAFE), University of Basilicata, Potenza, Italy; ^2^ Agenzia Lucana di Sviluppo e Innovazione in Agricoltura (ALSIA) Centro Ricerche Metapontum Agrobios, Metaponto, Italy

**Keywords:** scion-rootstock combinations, water stress, waterlogging tolerance, leaf gas exchanges, photosynthetic responses, affordable phenotyping, plant imaging, kiwifruit

## Abstract

**Introduction:**

Kiwifruit species have a relatively high rate of root oxygen consumption, making them very vulnerable to low root zone oxygen concentrations resulting from soil waterlogging. Recently, kiwifruit rootstocks have been increasingly used to improve biotic and abiotic stress tolerance and crop performance under adverse conditions. The aim of the present study was to evaluate morpho-physiological changes in kiwifruit rootstocks and grafting combinations under short-term waterlogging stress.

**Methods:**

A pot trial was conducted at the ALSIA PhenoLab, part of the Phen-Italy infrastructures, using non-destructive RGB and NIR image-based analysis and physiological measurements to identify waterlogging stress indicators and more tolerant genotypes. Three pot-grown kiwifruit rootstocks (‘Bounty 71,’ *Actinidia macrosperma*—B; ‘D1,’ *Actinidia chinensis* var. *deliciosa*—D; and ‘Hayward,’ *A. chinensis* var. *deliciosa*—H) and grafting combinations, with a yellow-fleshed kiwifruit cultivar (‘Zesy 002,’ *A. chinensis* var. *chinensis*) grafted on each rootstock (Z/B, Z/D, Z/H), were subjected to a control irrigation treatment (WW), restoring their daily water consumption, and to a 9-day waterlogging stress (WL), based on substrate saturation. Leaf gas exchange, photosynthetic activity, leaf temperature, RGB, and NIR data were collected during waterlogging stress.

**Results:**

Stomatal conductance and transpiration reached very low values (less than 0.05 mol m^−2^ s^−1^ and 1 mmol m^−2^ s^−1^, respectively) in both waterlogged D and H rootstocks and their grafting combinations. In turn, leaf temperature was significantly increased and photosynthesis was reduced (1–6 μmol m^−2^ s^−1^) from the first days of waterlogging stress compared to B rootstock and combination.

**Discussion:**

The B rootstock showed prolonged leaf gas exchange and photosynthetic activity, indicating that it can cope with short-term and temporary waterlogging and improve the tolerance of grafted kiwi vines, which showed a decrease in stomatal conductance 5 days after the onset of stress. Morphometric and colorimetric parameters from the image-based analysis confirmed the greater susceptibility of D and H rootstocks and their grafting combinations to waterlogging stress compared to B. The results presented confirm the role of physiological measurements and enhance that of RGB and NIR images in detecting the occurrence of water stress and identifying more tolerant genotypes in kiwifruit.

## 1 Introduction

Kiwifruit is a very important fruit crop in Italy, which is actually the world’s third-largest producer, with 24,040 ha and annual production of 523,120 tons (FAOSTAT, 2022), gaining commercial importance and playing a key role in kiwifruit trade dynamics (Mian et al., 2022a). Recently, the introduction of new commercially grown kiwifruit cultivars has increased in the yellow-fleshed category (Burdon et al., 2014), reducing the choice of the most popular green cultivars. Kiwifruit vines are particularly exposed and sensitive to climatic conditions (Cradock-Henry, 2017) and to biotic (Vanneste, 2017) and abiotic stresses [e.g., water stress (Xiloyannis et al., 2023) and salinity stress (Abid et al., 2020)]. In recent years, increased solar radiation and summer temperatures and irregular rainfall distribution, both exacerbated by climate change, have posed new climatic challenges for kiwifruit cultivation (Testolin and Ferguson, 2009). Research and selection of new genetic material (rootstocks and cultivars) is essential to meet the new challenges posed by changing environmental conditions, with an increase in extreme events and the emergence of stresses.

Since kiwifruit cultivation has received particular attention, physiological disorders have been observed in orchards around the world as a long-term consequence of flooding events (Reid et al., 1991). In Italy, kiwifruit physiological disorders have been reported since 2012 and have been referred to as the “kiwifruit vine decline syndrome” (KVDS), which is seriously threatening many cultivated areas, rapidly spreading across northern and central Italy (Bardi, 2020). Although a multifactorial origin of this syndrome has been suggested (Donati et al., 2020; Bardi et al., 2022), waterlogging and asphyxiating conditions represent triggering factors for the expression of KVDS (Bardi, 2020; Donati et al., 2020; Savian et al., 2020; Spigaglia et al., 2020; Sofo et al., 2022; Di Biase et al., 2023; Prencipe et al., 2023; Sofo et al., 2024). Waterlogging stress can be injurious or even lethal to crops as water saturates the root environment, reducing oxygen availability, affecting nutrient and water uptake, and promoting a shift to the anaerobic metabolism (Sairam et al., 2008), which lead to potential economic loss in fruit tree crops (Li Z. et al., 2021). Waterlogging conditions easily occur in kiwifruit orchards due to extreme meteorological events and improper and empirical irrigation management (Bardi, 2020; Sofo et al., 2024), exacerbated by heavy and poorly drained soils characterized by clay-loam to clay textures and low organic matter content (Domingo et al., 2002). Frequent or long-term waterlogging conditions also lead to a more or less gradual deterioration of soil structure and quality and the establishment of anoxic conditions (Sofo et al., 2022, 2024). Plant growth is significantly compromised by oxygen deficiency or lack, and roots only grow in small areas of more oxygenated soil, greatly reducing the potential for root exploration of the soil volume under aerated conditions (Sairam et al., 2008). Previous studies demonstrated that kiwifruit has a high-water requirement (Holzapfel et al., 2000) but, at the same time, is extremely vulnerable to waterlogging and low oxygen concentrations in the root zone, which have a detrimental effect on plant growth and stomatal activity (Savé and Serrano, 1986; Smith et al., 1989, 1990), being one of the most waterlogging intolerant fruit trees (Bai et al., 2019). Plant responses to waterlogging and oxygen deprivation involve a wide range of metabolic, hormonal, and morphological adaptation and physiological and antioxidative defense processes (Domingo et al., 2002; Sairam et al., 2008; Li Z. et al., 2021; Zahra et al., 2021). In fruit tree crops, resistance to waterlogging and consequent oxygen deficiency can be influenced by the characteristics of the rootstock (Domingo et al., 2002), which can activate several mechanisms to delay the abiotic stress-induced effects (Fullana-Pericàs et al., 2020). Changes in environmental factors due to global climate change and the specific physiological traits and anatomy of kiwifruit vines pose emphasis on accurate orchard management and suitable techniques to avoid reductions in kiwifruit production areas, which are severely threatened by the rapid spread of physiological decline, and to increase new plantings in different climate scenarios. In order to promote the expansion in regions with soil and other environmental challenging factors, grafting kiwifruit cultivars on rootstocks with resistance or tolerance characteristics to different soil stresses could be of great importance (Mian et al., 2022b). All available measures, from the adoption of the best agronomic practices to avoid the establishment of waterlogging conditions to the identification of tolerant rootstocks, should be implemented to address the current challenges. Although genetic and breeding programs have increased the number of cultivars in recent years (Mian et al., 2022a), few rootstocks are currently commercially available for kiwifruit propagation (Clearwater et al., 2004; Li D. et al., 2021). The main cultivated kiwifruit cultivars, from *Actinidia chinensis* var. *deliciosa* and *A. chinensis* var. *chinensis*, are typically grafted onto *A. chinensis* var. *deliciosa* seedlings for commercial planting (Friend et al., 2014; Li D. et al., 2021). Previous studies focused on the physiological responses of *Actinidia* genotypes to waterlogging stress, including some rootstocks used for kiwifruit cultivation, with the aim of identifying tolerant genotypes and describing waterlogging tolerance mechanisms (Li et al., 2020; Li Z. et al., 2021). Among these, *A. chinensis* var. *deliciosa* (cv ‘Hayward’) has been reported as a sensitive rootstock to waterlogging stress, showing a rapid decrease in stomatal conductance, transpiration, and net photosynthetic rate with severe damages to the leaves and root system a few days after waterlogging stress application (Li Z. et al., 2021; Kataoka et al., 2021; Beppu et al., 2022). In contrast, *Actinidia macrosperma* has a greater tolerance to waterlogging conditions, maintaining relatively high levels of leaf gas exchange and photosynthetic activity for a longer time under waterlogging stress, with rare leaf burn and defoliation processes (Beppu et al., 2022), whose tolerance has also been reported in the kiwifruit scion grafted on it (Kataoka et al., 2021). Although several studies have been carried out to evaluate the molecular and physiological responses to waterlogging, phenotyping for waterlogging tolerance has been poorly addressed (Langan et al., 2022). Image-based phenotyping methods allow the assessment of shoot and root traits, such as shoot biomass and morphometry, photosynthesis-related traits, root biomass and architecture, with both advantages and challenges to detect and improve the characterization of waterlogging tolerance among crop species (Langan et al., 2022). In the present research, a phenotyping approach based on the identification of both morphometric and colorimetric traits and indicators, accompanied by monitoring of physiological parameters, is used for the first time in kiwifruit to improve the assessment of waterlogging response. A short-term waterlogging experiment was conducted a) to assess the tolerance/susceptibility of different kiwifruit rootstocks to waterlogging stress based on physiological and phenotyping analysis, b) to evaluate the effect of the rootstock on the responses of the grafted yellow-fleshed kiwifruit cultivar to waterlogging, and c) to define a methodology integrating physiological and phenotyping indicators to screen for more tolerant genotypes to abiotic stress.

## 2 Materials and methods

### 2.1 Plant material and growth conditions

The experimental trial was conducted at the ALSIA ‘Metapontum Agrobios’ Research Centre in Metaponto (South Italy) during the 2023 growing season. A total of 30 two-year-old self-rooted vines of ‘Bounty 71’ (B) (*A. macrosperma*), ‘D1’ (D) (*A. chinensis* var. *deliciosa*), and ‘Hayward’ (H) (*A. chinensis* var. *deliciosa*) and 42 one-year-old scions of *A. chinensis* var. *chinensis* (cv ‘Zesy 002,’ also known as ‘G3’) grafted on each rootstock (Z/B, Z/D and Z/H) were used in the experiment. All kiwifruit vines were transplanted in March into 3 L pots filled with a mixture of acid peat and pumice (pH 6, bulk density 0.23 g cm^−3^, and 90% porosity). The vines were grown in an unheated and unconditioned greenhouse under natural light conditions and randomly distributed to reduce the effect of possible microclimatic variations in the greenhouse through spatial distribution. All vines were trained with a single main shoot and tied to a wooden stick support painted blue to allow image acquisition, segmentation, and subsequent data analysis. Vines were pruned to a similar leaf area of approximately 10 leaves each. Each pot was identified by a barcode to allow the platform to automatically read and identify the vines during image acquisition. From transplanting until the beginning of the experimental trial, all vines were fully irrigated according to evapotranspiration demands.

### 2.2 Experimental design and irrigation treatments

Before the beginning of the experimental trial, the reference pot weight, corresponding to the field capacity (FC), was determined by fully watering each pot and then allowing the excess water to drain until a stable pot weight was reached. On 10 July, five vines from each rootstock and seven from each scion–rootstock combination were subjected to waterlogging stress, while an equal number of each rootstock and scion–rootstock combination continued to be optimally irrigated. In particular, the well-watered (WW) vines were optimally irrigated by maintaining the soil water content of the pots close to FC, replenishing water lost during the day by automatically weighing the pots three times a day and restoring the reference weight at FC. The waterlogging (WL) treatment was carried out by manually overwatering the vines. For nine consecutive days of waterlogging stress (referred to as days after waterlogging, DAW), the substrate was kept continuously saturated. Each vine was placed in a plastic container filled with water to a height of 1/3 of the pot to simulate soil conditions, which are one of the factors that trigger physiological decline in the field.

### 2.3 Leaf gas exchange measurements

Stomatal conductance (gs) and transpiration (E) were measured during the day at approximately 3-h intervals for rootstocks (9:00, 11:00, 14:00, and 16:00 h, solar time) and scion–rootstock combinations (8:00, 10:00, 13:00, and 15:00 h, solar time) at 1, 2, 3, and 4 DAW and at the hours of maximum efficiency (10:00–12:00 h) at 5, 7, and 9 DAW using a portable handheld LI-600 porometer system integrated with a fluorometer (Li-Cor Biosciences, Lincoln, USA). Photosynthesis (A), internal CO_2_ concentration (Ci), and leaf temperatures (T_leaf_) were measured during the day at approximately 3-h intervals for rootstocks (9:00, 11:00, 14:00, and 16:00 h, solar time) and scion–rootstock combinations (8:00, 10:00, 13:00, and 15:00 h, solar time) at 1, 2, 3, and 4 DAW using a portable photosynthesis system Li-Cor 6800 (Li-Cor, Inc., Lincoln, NE, USA). Leaf gas exchange measurements were taken at an ambient (CO_2_) of 400 µmol mol^−1^, with temperature and external ambient photosynthetic photon flux density (PPFD) maintained at the prevailing external environmental conditions and the operating flow rate set at 500 μmol s^−1^. To minimize the potential effects of leaf position and developmental age, all the gas exchange measurements were taken on a fully expanded and exposed leaf, with no visual symptoms of stress, from the mid-shoot region of four randomly selected vines for each irrigation treatment, rootstock, and scion–rootstock combination. The intrinsic water-use efficiency was calculated as WUEi = A/gs (Bellasio, 2023).

### 2.4 High-throughput plant phenotyping

Phenotyping measurements, based on the morphological and physiological characterization of the vines, were carried out non-destructively through plant imaging. Five vines per irrigation treatment from each rootstock and scion–rootstock combination were imaged at 1, 2, 3, 4, 7, and 9 DAW using a Scanalyzer 3D plant phenotyping platform (LemnaTec GmbH, Aachen, Germany), which is part of the Phen-Italy platform located at ALSIA ‘Metapontum Agrobios.’ The vines were automatically transported by a conveyor belt into the two imaging chambers with two types of illumination, broad-spectrum halogen lighting for the near-infrared (NIR) chamber and fluorescent white lighting for the visible light (RGB) chamber, and identified using a barcode and RFID tracking system. The NIR chamber was equipped with NIR cameras operating in the 900–1,700 wavelength range (VDS Vosskühler GmbH NIR-300P), and the images were used to assess the water content of plant tissues, as reported in Petrozza et al. (2014). The visible light chamber for RGB imaging was equipped with cameras with a resolution of approximately 2 megapixels (Basler scA1600-28gc). The images were used to evaluate the health status and stress responses of the vines by analyzing color (i.e., green color corresponding to healthy tissue and degree of yellowing indicative of chlorotic tissues) and morphometric parameters such as projected shoot area and solidity. Three images were acquired from each chamber, one from the top of the plant (top view, TV) and two from the lateral sides at an orthogonal angle (0° and 90° side view, SV), to provide an average plant area correction for possible leaf overlap and then a robust representation of the total plant area (Golzarian et al., 2011). NIR acquisitions were taken at 2, 3, and 9 DAW and simultaneously with RGB images.

### 2.5 Image analysis

Image segmentation, which separated the plant from the background by creating sufficient contrast, and subsequent image analysis were performed using Python v3.9 and PlantCV v3.11 open-source software (Fahlgren et al., 2015). In particular, the sum of the number of pixels corresponding to the plant object area, derived from the RGB images of the two orthogonal SVs and the TV, was converted into cm^2^ using a calibration factor (Golzarian et al., 2011) and referred to as the projected shoot area (PSA), determined following a procedure similar to that reported by Genangeli et al. (2023):


(1) PSA=Area(TV)+Area(0°SV)+Area(90°SV)

Where the result is the sum of the three target plant areas from the top and from the side views expressed in cm^2^.

How much of the hull area of the plant is covered by the leaves was identified through the solidity (S) and calculated as the ratio of the plant pixel area to the convex hull shape pixel area containing all plant pixels from plant images (Petrozza et al., 2023).

RGB images were then analyzed with the hue component, which corresponds to an angular position around a central point, scaled in degrees from 0° to 360°, with different degrees indicating different colors. Leaf and total plant color were obtained by calculating the weighted mean value from the histogram of the hue channel in the hue saturation value (HSV) color space, resulting in a value from 0° to 360° (Petrozza et al., 2023). Usually, leaf color is included in the hue range from 120° (green—healthy tissue) to 60° (yellow—chlorotic tissue). The component hue (in degrees) was then used to calculate the green and the greener fractions as the sum of green pixels included in the hue angle ranges of 60° ≤ hue ≤ 180° and 80° ≤ hue ≤ 180°, respectively. These fractions were used to calculate the senescence index (SI), which indicates the degree of plant senescence, as follows (Genangeli et al., 2023):


(2) SI=(GAS-GerAS)/GAS

where GAS is the green area calculated from the side view, corresponding to the sum of the pixels in the hue angular region from 60° to 180°. GerAS is the greener area calculated from the side view, corresponding to the sum of the pixels in the hue angular region from 80° to 180°.

NIR images were acquired from a side view. The final results were expressed as NIR intensities obtained at the typical water absorption wavelength of 1,450 nm as previously reported in Kim et al. (2020). An increased level of absorption determines a reduced level of reflectance in the NIR spectrum, corresponding to an increased water content of plant tissues. Vine tissues with a high-water content showed a low NIR intensity.

### 2.6 Data analysis

Statistical analysis was performed using the R software (4.3.3 version, R Foundation for Statistical Computing, Vienna, Austria), and data were plotted using SigmaPlot 15.0 (Systat Software, Inc., San Jose, CA, USA). Both physiological and phenotyping measurements were reported as mean values and standard error of the means (± SE). The normal distribution of the data was evaluated both visually (QQ-plot) and statistically (Shapiro–Wilk normality test). Levene’s test was used to check for homogeneity of variances. Two-way ANOVA analysis was used to determine the effects of irrigation treatments and cultivars on physiological and phenotypic parameters at each DAW. One-way ANOVA was used to examine the differences between irrigation treatments within each cultivar at each DAW. Multiple comparisons of means between irrigation treatments and cultivars within each DAW were made using Tukey’s honest significant difference (HSD) *post-hoc* test with *p*-values <0.05 considered significant.

## 3 Results

### 3.1 Effect of waterlogging on the photosynthetic capacity

**Figure 1:**
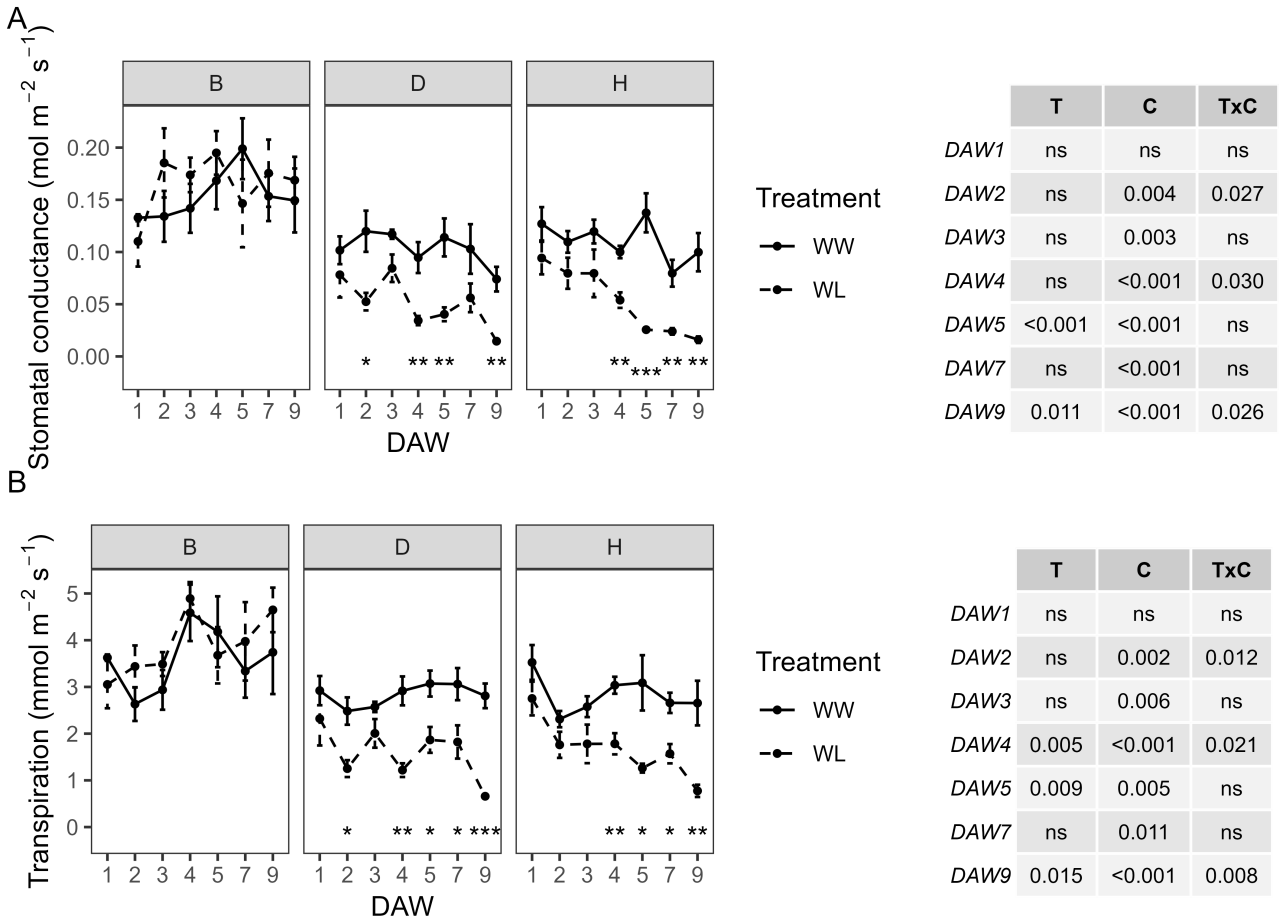
Effects of waterlogging stress on **(A)** stomatal conductance (gs) and **(B)** transpiration (E) of different rootstocks (B, D, H) during waterlogging days (11:00 h). Two-way ANOVA *p*-values are shown in the tables for both gs and E and for each DAW. Asterisks denote significant differences according to one-way ANOVA between WW and WL for each parameter and rootstock. Data shown are means ± standard error of the means (*n* = 4). T, treatment effects; C, cultivar effects; ns, not significant at p≥0.05.

The WW vines showed a similar behavior for both the D and H rootstocks, with values of gs approximately 0.1–0.14 mol m^−2^ s^−1^ and E approximately 2.3–3.5 mmol m^−2^ s^−1^. The B rootstock was characterized by slightly higher values, peaking at 0.2 mol m^−2^ s^−1^ and 4.6 mmol m^−2^ s^−1^, respectively. A significant effect of cultivar was observed throughout the experiment for gs and E, with a lesser treatment effect appearing from DAW 5 and 4, respectively. WL vines of the D and H rootstocks showed a significant and progressive decrease in gs and E from DAW 4. Instead, vines of the B rootstock subjected to waterlogging showed gs and E values similar to those of WW vines, with no significant differences on all days of stress (**Figure 1**). In particular, significant reductions of approximately 80%–85% in gs and 70%–75% in E were observed in WL vines of the D and H rootstocks at the end of the waterlogging stress experiment (DAW 9) (**Figures 1A, B**).

**Figure 2:**
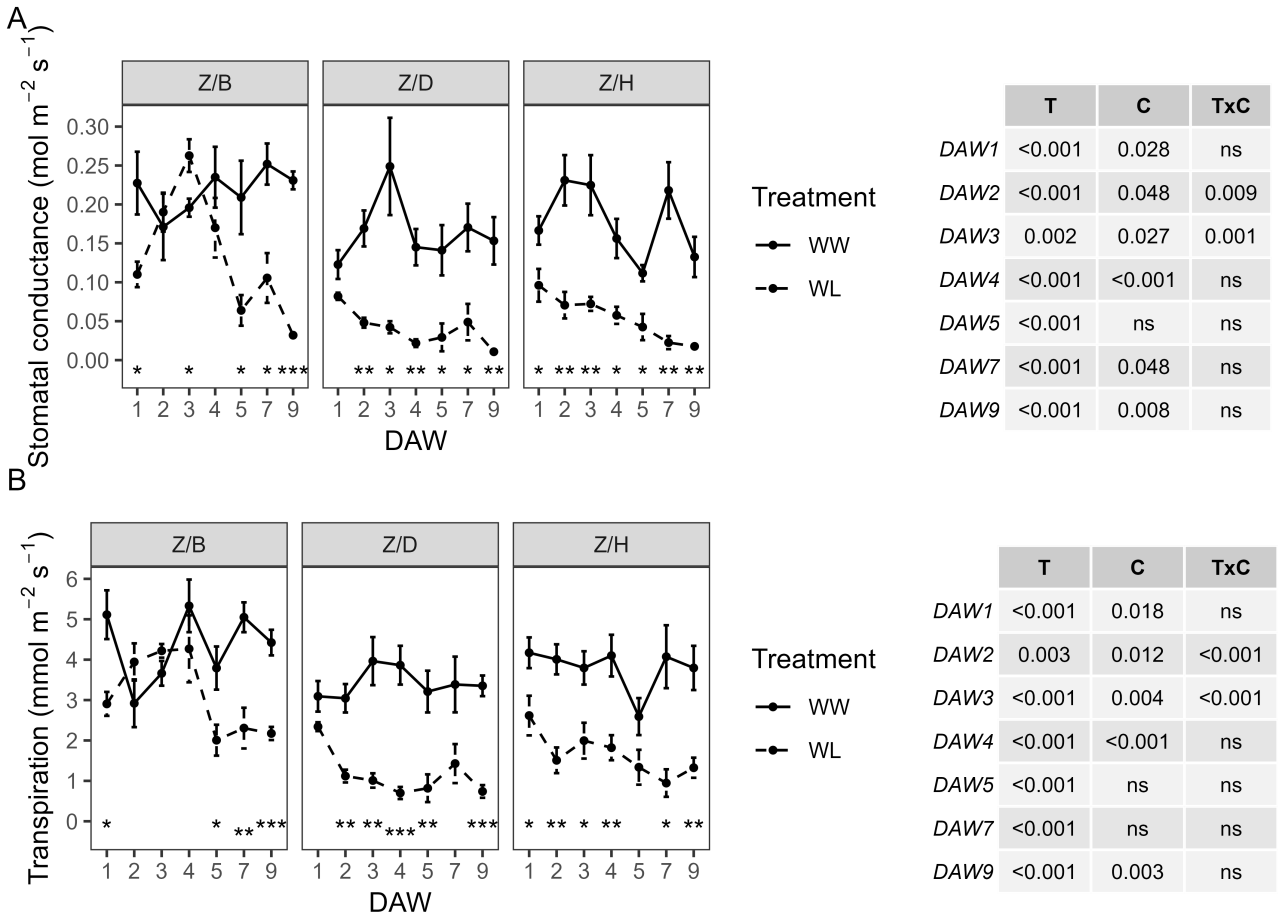
Effects of waterlogging stress on **(A)** stomatal conductance (gs) and **(B)** transpiration (E) of different scion–rootstock combinations (Z/B, Z/D, Z/H) during waterlogging days (10:00 h). Two-way ANOVA *p*-values are shown in the tables for both gs and E and for each DAW. Asterisks denote significant differences according to one-way ANOVA between WW and WL for each parameter and scion–rootstock combination. Data shown are means ± standard error of the means (*n* = 4). T, treatment effects; C, cultivar effects, ns, not significant at p≥0.05.

Z/D and Z/H combinations showed a similar behavior in gs and E, both under well-watered and stressed conditions. The Z/B combination was characterized by slightly higher values in WW vines compared to the other combinations and a slower decline in physiological parameters in WL vines compared to WW vines (**Figure 2**). A consistently significant (*p* < 0.001) effect of the waterlogging treatment was observed throughout the experiment from the start of stress application for both physiological indicators. Minimum gs values, less than 0.05 mol m^−2^ s^−1^, were reached in WL vines of all combinations at the end of the waterlogging stress experiment (DAW 9). In the Z/B combination, gs of WL vines remained within the range of control values from DAW 1 to DAW 4, then started to decrease significantly at DAW 5. A minimum of 0.03 mol m^−2^ s^−1^ was reached in the Z/B combination after 9 days of waterlogging stress, with a reduction of approximately 86% compared to WW vines. For the Z/D and Z/H combinations, WL vines already had gs values below 0.05 mol m^−2^ s^−1^ from DAW 2 and DAW 5, respectively. Reductions in gs of approximately 93% and 87% compared to WW vines were achieved in Z/D and Z/H combinations, respectively, at the end of the waterlogging stress experiment (**Figure 2A**). Minimum E values of less than 1 mmol m^−2^ s^−1^ were reached in WL vines of the Z/D and Z/H combinations between DAW 7 and DAW 9, while minimum values of approximately 2 mmol m^−2^ s^−1^ were reached in the Z/B combination from DAW 5. At the end of the experiment, E was reduced by approximately 51%, 78%, and 65% in the Z/B, Z/D, and Z/H combinations, respectively (**Figure 2B**).

**Figure 3:**
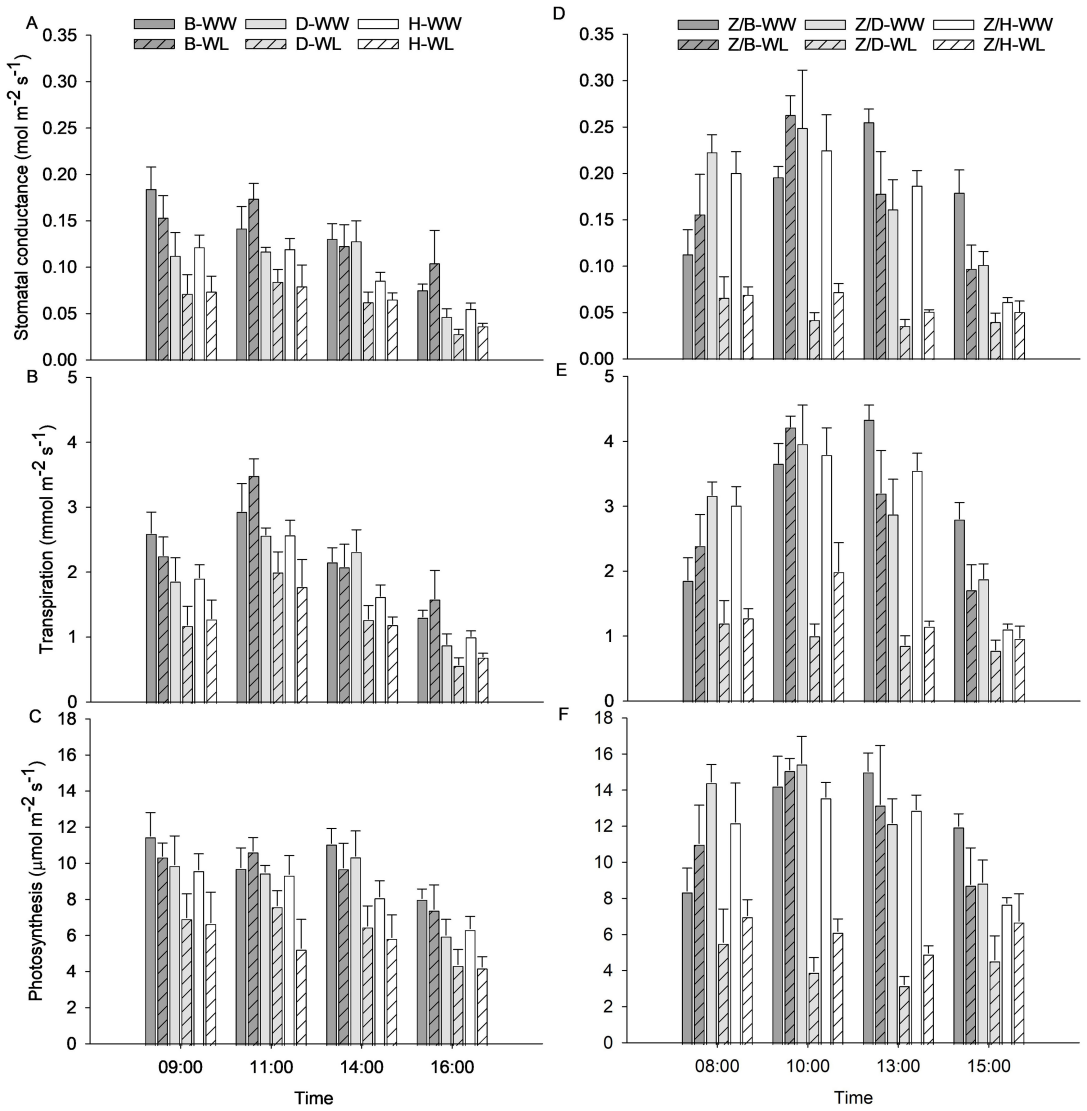
Diurnal trends of **(A, D)** stomatal conductance (gs), **(B, E)** transpiration (E), and **(C, F)** photosynthesis (A) of the different rootstocks (B, D, H) and scion–rootstock combinations (Z/B, Z/D, Z/H) 3 days after waterlogging stress. Data presented are means ± standard error of the means (*n* = 4).

Diurnal trends of gs, E, and A measured 3 days after waterlogging stress are shown in **Figure 3** to highlight the physiological behavior of the WW and WL vines of rootstocks and scion–rootstock combinations throughout the day. The physiological activity of the WW rootstocks appeared to be less intense than that of the combinations, with slightly lower values for gs, E, and A. In addition, the rootstocks appeared to be more resilient to waterlogging stress than the combinations, which showed a more pronounced decrease in physiological parameters under waterlogging stress. Stomatal conductance was higher in the morning hours and started to decrease from the early afternoon in WW vines of all rootstocks (**Figure 3A**) and Z/D and Z/H combinations (**Figure 3D**). In the Z/B combination, gs reached higher values in the early afternoon (**Figure 3D**). The WL vines of the D and H rootstocks and the Z/D and Z/H combinations showed an almost constant gs throughout the day, reaching values greatly reduced compared to the WW vines. On the other hand, the WL vines of the B rootstock and the Z/B combination behaved similarly to the WW vines, and only a slight reduction in gs was observed in the afternoon hours for the Z/B combination (**Figures 3A, D**). The E of the WW vines followed the gs, increasing slightly in the middle hours of the morning, when light and other environmental parameters become more demanding, and then starting to decrease from the early afternoon. The WL vines of the Z/D combination had the lowest E values, which were almost constant throughout the day (**Figures 3E**). The A showed a similar behavior to the gs and was strongly affected by waterlogging stress (**Figures 3C, F**). In particular, greater reductions in A were observed in WL vines of the D and H rootstocks (**Figure 3C**) and the Z/D and Z/H combinations (**Figure 3F**), while WL vines of the B rootstock and the Z/B combination maintained values similar to those of WW vines.

The D and H rootstocks had similar A values, while slightly higher values were generally observed in the B rootstock, with significant differences between rootstocks at DAW 2. As reported in **Table 1**, A of the D and H rootstocks gradually decreased with prolonged waterlogging exposure. A significant reduction in A due to waterlogging stress was observed in the D and H rootstocks at DAW 4, with values reduced by approximately 63% and 47%, respectively, compared to WW rootstocks. The B rootstock was unaffected by waterlogging, with values similar to those of the WW rootstocks (**Table 1**). All WL rootstocks showed no changes in Ci values during the first 4 days of waterlogging stress, suggesting that the stress had not yet affected the balance between CO_2_ intake (controlled by stomatal conductance) and CO_2_ fixation (controlled by photosynthetic capacity). However, significant differences in Ci were observed between B and D and H rootstocks at DAW 3 and DAW 4, with the B rootstock showing slightly higher values, which may be associated with the higher gs (**Figure 1A**) and then CO_2_ intake (**Table 1**). The WUEi was not affected by waterlogging stress during the first 4 days of the experiment for any of the rootstocks. Lower WUEi values were observed for the B rootstock compared to the D and H rootstocks throughout the 4 days, with significant differences at DAW 3 and DAW 4, suggesting a lower water use efficiency for the B rootstock (**Table 1**). In particular, high gs values in the B rootstock resulted in increased CO_2_ intake through the open stomata, but at the same time, more water was lost through transpiration. Higher values of Ci indicated the inability of the photosynthetic apparatus to use the increased CO_2_ effectively, leading to an accumulation of CO_2_ in the leaf, and lower values of WUEi indicated that the vines used more water (high gs, E) to assimilate the same amount of carbon. A significant increase in T_leaf_ due to waterlogging stress was observed at DAW 4, with values increased by approximately 2% and 1% in WL vines of D and H rootstocks, respectively, compared to WW rootstocks. The T_leaf_ of the B rootstock was not affected by waterlogging and showed values in the range of the WW rootstock (**Table 1**). Lower values of T_leaf_ were generally observed for the B rootstock, followed by the H and D rootstocks, suggesting that higher gs and E increased the cooling effect of the leaves in the B rootstock (**Figures 1A, B**).

**Table 1:** Effects of waterlogging stress on photosynthesis (A), internal CO_2_ concentration (Ci), intrinsic water use efficiency (WUEi), and leaf temperature (T_leaf_) in different rootstocks (B, D, H) during the first 4 days of the experiment (11:00 h).

	Treatment	Rootstock			Treatment	Rootstock			Treatment
		B	D	H		B	D	H	
		A (µmol m^−2^ s^−1^)				WUEi (μmol CO_2_ mol^−1^ H_2_O)			
DAW 1	Well-watered	10.55 ± 0.34	8.46 ± 0.56	10.09 ± 1.19	9.70 ± 0.49	79.44 ± 2.06	85.69 ± 6.39	81.04 ± 7.90	82.06 ± 3.23
	Waterlogged	8.01 ± 1.45	7.03 ± 1.72	8.43 ± 1.31	7.82 ± 0.80	74.43 ± 3.05	92.93 ± 3.86	90.58 ± 5.04	85.98 ± 3.26
	Rootstock	9.28 ± 0.84	7.75 ± 0.88	9.26 ± 0.88	–	76.94 ± 1.95	89.31 ± 3.72	85.81 ± 4.70	–
DAW 2	Well-watered	9.73 ± 1.03^AB^	9.47 ± 1.23^AB^	7.96 ± 0.73^AB^	9.05 ± 0.58	75.69 ± 6.54	80.46 ± 3.69	74.02 ± 7.51	76.72 ± 3.31
	Waterlogged	11.44 ± 1.78^A^	4.65 ± 0.89^B^	6.14 ± 1.37^AB^	7.41 ± 1.14	63.03 ± 3.56	86.82 ± 7.61	74.74 ± 6.81	74.86 ± 4.38
	Rootstock	10.58 ± 1.01^a^	7.06 ± 1.15^b^	7.05 ± 0.80^b^	–	69.36 ± 4.19	83.64 ± 4.10	74.38 ± 4.70	–
DAW 3	Well-watered	9.76 ± 1.10	9.51 ± 0.38	9.38 ± 1.04	9.55 ± 0.47	72.12 ± 7.40	81.34 ± 2.74	78.43 ± 4.39	77.30 ± 2.96
	Waterlogged	10.67 ± 0.76	7.64 ± 0.84	5.29 ± 1.60	7.87 ± 0.89	61.81 ± 1.64	93.46 ± 8.33	66.10 ± 6.15	73.79 ± 5.28
	Rootstock	10.21 ± 0.64	8.58 ± 0.55	7.34 ± 1.18	–	66.97 ± 4.01^b^	87.40 ± 4.66^a^	72.26 ± 4.20^ab^	–
DAW 4	Well-watered	11.43 ± 1.26^A^	8.58 ± 1.12^AB^	8.48 ± 0.40^AB^	9.50 ± 0.67^a^	70.84 ± 6.87	91.73 ± 3.97	85.18 ± 3.42	82.58 ± 3.70
	Waterlogged	11.78 ± 1.16^A^	3.16 ± 0.59^C^	4.48 ± 0.85^BC^	6.47 ± 1.24^b^	70.00 ± 3.57	89.24 ± 7.97	82.52 ± 6.87	77.59 ± 4.94
	Rootstock	11.61 ± 0.79^a^	5.87 ± 1.18^b^	6.48 ± 0.87^b^	–	65.92 ± 4.04^b^	90.49 ± 4.15^a^	83.85 ± 3.59^a^	–
		Ci (µmol mol^−1^)				T_leaf_ (°C)			
DAW 1	Well-watered	260.92 ± 3.56	253.60 ± 9.44	258.74 ± 13.26	257.76 ± 5.11	32.45 ± 0.03	32.88 ± 0.13	32.59 ± 0.07	32.64 ± 0.07
	Waterlogged	271.91 ± 3.75	243.81 ± 4.20	245.61 ± 8.14	253.78 ± 4.89	32.61 ± 0.21	33.03 ± 0.11	32.91 ± 0.18	32.85 ± 0.10
	Rootstock	266.42 ± 3.17	248.71 ± 5.13	252.18 ± 7.62	–	32.53 ± 0.10^b^	32.95 ± 0.08^a^	32.75 ± 0.11^ab^	–
DAW 2	Well-watered	272.33 ± 9.54	264.71 ± 5.02	277.02 ± 12.44	271.35 ± 5.19	32.34 ± 0.09	32.59 ± 0.17	32.48 ± 0.18	32.47 ± 0.08
	Waterlogged	289.96 ± 4.96	260.60 ± 13.06	278.00 ± 12.05	276.19 ± 6.64	32.24 ± 0.18	33.07 ± 0.16	32.73 ± 0.21	32.68 ± 0.14
	Rootstock	281.15 ± 5.99	262.66 ± 6.52	277.51 ± 8.02	–	32.29 ± 0.10^b^	32.83 ± 0.14^a^	32.61 ± 0.14^ab^	–
DAW 3	Well-watered	277.25 ± 10.72	262.75 ± 4.66	267.71 ± 7.82	269.23 ± 4.61	32.50 ± 0.09	32.65 ± 0.21	32.47 ± 0.09	32.54 ± 0.08
	Waterlogged	292.33 ± 1.83	245.65 ± 12.69	292.35 ± 10.33	276.78 ± 8.29	32.41 ± 0.05	32.93 ± 0.04	32.41 ± 0.24	32.58 ± 0.10
	Rootstock	284.79 ± 5.79^a^	254.20 ± 7.04^b^	280.03 ± 7.59^a^	–	32.45 ± 0.05	32.79 ± 0.11	32.44 ± 0.12	–
DAW 4	Well-watered	272.39 ± 9.82	242.75 ± 6.09	253.49 ± 5.45	256.21 ± 5.34	33.06 ± 0.12	33.74 ± 0.19	33.46 ± 0.08	33.42 ± 0.11^b^
	Waterlogged	288.45 ± 6.15	252.79 ± 13.08	262.44 ± 11.35	267.89 ± 7.16	32.98 ± 0.07	34.31 ± 0.08	33.77 ± 0.17	33.69 ± 0.17^a^
	Rootstock	280.42 ± 6.16^a^	247.77 ± 6.94^b^	257.97 ± 6.07^b^	–	33.02 ± 0.06^c^	34.03 ± 0.14^a^	33.61 ± 0.11^b^	–

The means of each rootstock are compared within the same row, the means of each irrigation treatment are compared within the same column, and the interaction between all values is assessed using Tukey’s honest significant difference (HSD) comparison test (*p* < 0.05). Different lowercase letters indicate significant differences in the means of each treatment and each rootstock, while different uppercase letters indicate significant differences of the interaction (*n* = 4 ± SE). Note that letters were not reported when there were no significant differences.

Waterlogging caused significant changes in A of the scion–rootstock combinations during all the first 4 days of the experiment. In particular, the reductions in A were more pronounced in the Z/D and Z/H combinations, where A decreased from DAW 1, and with prolonged waterlogging exposure, by approximately 89% and 52%, respectively, at DAW 4 (**Table 2**). Higher values of A were generally observed in the Z/B combination, with significant differences at DAW 3 and DAW 4, reinforced by the little or no effect of waterlogging on the Z/B combination compared to Z/D and Z/H combinations. Slight reductions in A were observed in the Z/B combination only at DAW 1, consistent with the response in gs and E (**Figures 2A, B**), and at DAW 4, but with no significant differences. The Ci values were significantly lower in the WL treatment at DAW 1, consistent with the reductions in gs and E observed in the combinations (**Figures 2A, B**). This suggests that lower gs affected CO_2_ intake, leading to a decrease in Ci, which in turn limited photosynthetic activity due to less CO_2_ available for fixation (**Table 2**). This tendency of Ci is maintained in the Z/D and Z/H combinations until DAW 3 and DAW 4, respectively, supporting the occurrence of moderate water stress during which the WL vines initially showed a decrease in Ci due to partial stomatal closure (**Figure 2A**). After 4 days of waterlogging stress, an inverse trend for Ci was observed in the WL vines of the Z/D combination, which showed significantly higher values compared to the WW vines. At this time, a more severe water stress condition occurred, as indicated by the rapid decrease in A (equal to 1.30 μmol m^−2^ s^−1^) and the strong impairment of the photosynthetic apparatus, which was no longer able to fix CO_2_, leading to an increased Ci concentration. The Z/B combination showed no changes in Ci values for the remaining days (DAW 2–4), suggesting that the stress had not yet affected the balance between CO_2_ intake and CO_2_ fixation, as supported by the still control values of gs and A (**Figure 2A**; **Table 2**). Different from the rootstocks, the WUEi of the rootstock–scion combinations was affected by waterlogging stress. Higher values were observed in the Z/D and Z/H combinations under waterlogging until DAW 3 and DAW 4, respectively, suggesting a more efficient use of water from water lost through transpiration (low gs) and carbon assimilation. An inverse trend, as for Ci, was also found for WUEi 4 days after waterlogging stress in the Z/D combination, which decreased, confirming the severe impairment of the photosynthetic apparatus (**Table 2**). For the Z/B combination, higher WUEi values were observed in the WL vines compared to the WW vines on DAW 1 and DAW 4, confirming the observations related to other parameters on these days and suggesting that DAW 4 represented the beginning of the stress response for the Z/B combination, although no significant differences were already observed. Higher T_leaf_ was found in the WL treatment compared to the WW treatment on all 4 days of waterlogging stress. In particular, T_leaf_ increased progressively in Z/D and Z/H combinations under waterlogging from DAW 2, reaching increases of approximately 4% and 2%, respectively, compared to the WW vines at DAW 4. The Z/B combination generally showed lower values of T_leaf_ compared to other combinations, and WL vines maintained values similar to those of the WW vines during the first 4 days of waterlogging stress (**Table 2**).

**Table 2:** Effects of waterlogging stress on photosynthesis (A), internal CO_2_ concentration (Ci), intrinsic water use efficiency (WUEi), and leaf temperature (T_leaf_) in different scion–rootstock combinations (Z/B, Z/D, Z/H) during the first 4 days of waterlogging (10:00 h).

	Treatment	Scion–rootstock combination			Treatment	Scion–rootstock combination			Treatment
		Z/B	Z/D	Z/H		Z/B	Z/D	Z/H	
		A (µmol m^−2^ s^−1^)				WUEi (μmol CO_2_ mol^−1^ H_2_O)			
DAW 1	Well-watered	13.63 ± 1.43	10.05 ± 1.52	12.58 ± 1.10	12.09 ± 0.84^a^	62.28 ± 4.79	81.86 ± 2.20	76.50 ± 4.66	73.55 ± 3.27^b^
	Waterlogged	8.25 ± 0.72	7.72 ± 0.71	8.63 ± 1.49	8.20 ± 0.56^b^	77.40 ± 6.11	93.68 ± 3.48	92.76 ± 4.62	87.95 ± 3.39^a^
	Combination	10.94 ± 1.26	8.89 ± 0.89	10.61 ± 1.14	–	69.84 ± 4.59^b^	87.77 ± 2.93^a^	84.63 ± 4.32^a^	–
DAW 2	Well-watered	12.79 ± 1.46^AB^	13.41 ± 1.55^A^	14.69 ± 1.28^A^	13.63 ± 0.79^a^	90.81 ± 24.91	80.51 ± 4.45	65.96 ± 6.12	79.09 ± 8.43
	Waterlogged	11.61 ± 1.09^AB^	5.32 ± 0.94^C^	7.46 ± 1.25^BC^	8.13 ± 0.98^b^	62.06 ± 3.84	109.06 ± 7.69	113.08 ± 14.62	94.73 ± 8.65
	Combination	12.20 ± 0.87	9.36 ± 1.75	11.07 ± 1.60	–	76.44 ± 12.87	94.78 ± 6.78	89.52 ± 11.54	–
DAW 3	Well-watered	14.25 ± 1.62^A^	15.48 ± 1.50^A^	13.61 ± 0.82^A^	14.45 ± 0.75^a^	60.52 ± 3.04	68.06 ± 7.85	64.10 ± 7.53	64.23 ± 3.53^b^
	Waterlogged	15.12 ± 0.64^A^	3.96 ± 0.76^B^	6.17 ± 0.69^B^	8.42 ± 1.50^b^	58.22 ± 3.29	95.65 ± 8.56	87.95 ± 11.03	80.60 ± 6.51^a^
	Combination	14.68 ± 0.82^a^	9.72 ± 2.31^b^	9.89 ± 1.49^b^	–	59.37 ± 2.12^b^	81.85 ± 7.49^a^	76.02 ± 7.65^ab^	–
DAW 4	Well-watered	14.04 ± 1.60^A^	11.62 ± 0.94^AB^	11.99 ± 1.36^AB^	12.55 ± 0.76^a^	62.29 ± 5.90^B^	82.94 ± 5.88^AB^	79.21 ± 5.87^AB^	74.81 ± 4.10
	Waterlogged	11.95 ± 2.30^AB^	1.30 ± 0.64^C^	5.71 ± 0.85^BC^	6.32 ± 1.52^b^	74.60 ± 7.48^AB^	50.23 ± 14.68^B^	102.00 ± 7.66^A^	75.61 ± 8.41
	Combination	13.00 ± 1.35^a^	6.46 ± 2.02^b^	8.85 ± 1.40^b^	–	68.45 ± 4.98^b^	66.58 ± 9.58^b^	90.61 ± 6.21^a^	–
		Ci (µmol mol^−1^)				T_leaf_ (°C)			
DAW 1	Well-watered	284.96 ± 6.14	259.02 ± 4.54	263.45 ± 7.92	269.15 ± 4.76^a^	32.10 ± 0.27	32.29 ± 0.13	32.39 ± 0.14	32.26 ± 0.11^b^
	Waterlogged	267.84 ± 9.27	241.47 ± 6.39	243.04 ± 5.59	250.78 ± 5.26^b^	32.44 ± 0.25	32.79 ± 0.09	32.52 ± 0.14	32.58 ± 0.10^a^
	Combination	276.40 ± 6.08^a^	250.25 ± 4.92^b^	253.25 ± 5.92^b^	–	32.27 ± 0.18	32.54 ± 0.12	32.45 ± 0.09	–
DAW 2	Well-watered	245.23 ± 38.35	260.34 ± 6.63	281.11 ± 9.18	262.23 ± 12.85	31.66 ± 0.11^B^	31.90 ± 0.07^AB^	31.88 ± 0.25^AB^	31.81 ± 0.09^b^
	Waterlogged	285.92 ± 7.35	225.25 ± 13.02	216.29 ± 22.48	242.49 ± 12.38	31.30 ± 0.14^B^	32.57 ± 0.20^A^	32.46 ± 0.14^A^	32.11 ± 0.19^a^
	Combination	265.58 ± 19.64	242.80 ± 9.47	248.70 ± 16.63	–	31.48 ± 0.11^b^	32.24 ± 0.16^a^	32.17 ± 0.17^a^	–
DAW 3	Well-watered	293.59 ± 4.27	277.18 ± 10.75	285.97 ± 12.12	285.58 ± 5.44	31.57 ± 0.16^C^	31.93 ± 0.23^BC^	31.87 ± 0.11^BC^	31.79 ± 0.10^b^
	Waterlogged	293.78 ± 5.13	256.50 ± 10.89	254.25 ± 15.28	268.18 ± 8.02	31.69 ± 0.08^BC^	33.17 ± 0.35^A^	32.56 ± 0.18^AB^	32.47 ± 0.22^a^
	Combination	293.69 ± 3.09^a^	266.84 ± 8.09^b^	270.11 ± 10.84^ab^	–	31.63 ± 0.09^b^	32.55 ± 0.30^a^	32.22 ± 0.16^a^	–
DAW 4	Well-watered	284.13 ± 8.31^AB^	253.97 ± 8.15^B^	259.44 ± 8.19^AB^	265.84 ± 5.84	32.01 ± 0.18^C^	32.43 ± 0.10^BC^	32.65 ± 0.16^BC^	32.36 ± 0.11^b^
	Waterlogged	266.88 ± 9.81^AB^	315.88 ± 23.51^A^	230.97 ± 11.83^B^	271.24 ± 13.48	32.31 ± 0.17^C^	33.82 ± 0.25^A^	33.19 ± 0.18^AB^	33.11 ± 0.21^a^
	Combination	275.50 ± 6.78^ab^	284.92 ± 16.42^a^	245.21 ± 8.56^b^	–	32.16 ± 0.13^b^	33.12 ± 0.29^a^	32.92 ± 0.15^a^	–

The means of each scion–rootstock combination are compared within the same row, the means of each irrigation treatment are compared within the same column, and the interaction between all values is assessed using Tukey’s honest significant difference (HSD) comparison test (*p* < 0.05). Different lowercase letters indicate significant differences in the means of each treatment and each scion–rootstock combination, while different uppercase letters indicate significant differences of the interaction (*n* = 4 ± SE). Note that letters were not reported when there were no significant differences.

**Figure 4:**
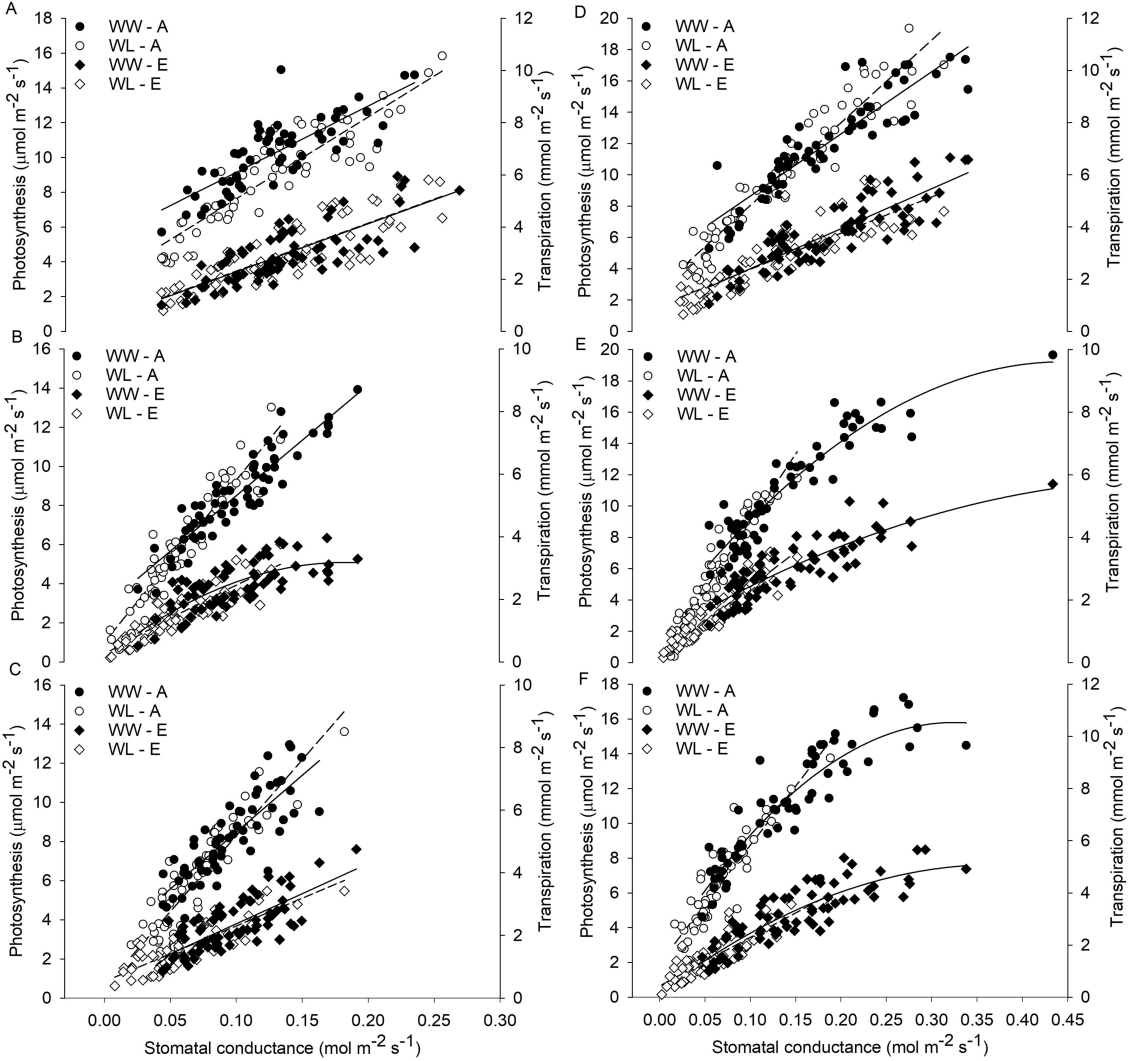
Relationships of stomatal conductance (gs) with photosynthesis (A) and transpiration rate (E) in waterlogged (WL) and well-watered (WW) vines for each rootstock **(A)** ‘Bounty71’ (B), **(B)** ‘D1’ (D), **(C)** ‘Hayward’ (H) and scion–rootstock combination **(D)** ‘Zesy/Bounty71’ (Z/B), **(E)** ‘Zesy/D1’ (Z/D), and **(F)** ‘Zesy/Hayward’ (Z/H). Data of all the vines collected during the 9-day waterlogging experiment are shown as individual points.

Relationships (linear or quadratic) between A and gs, as well as between E and gs, were found in both WL and WW vines of different rootstocks and scion–rootstock combinations (**Figure 4**). The results indicated that the reduction in gs could be related to a decrease in E and A activity of the vines, which differed according to the cultivar and treatment. No substantial differences in the relationships between A and gs and between E and gs were observed between WW and WL vines of the B rootstock (**Figure 4A**) and the Z/B combination (**Figure 4D**), indicating a similar behavior between treatments within the B cultivar. The relationships obtained for the D and H rootstocks showed very similar behavior of these rootstocks to waterlogging stress (**Figures 4B, C**). The WW vines of the different combinations showed similar relationships between A and gs and between E and gs, whereas the WL vines showed a different behavior (**Figures 4D–F**).

### 3.2 RGB image-based morphometric and colorimetric parameters

**Figure 5:**
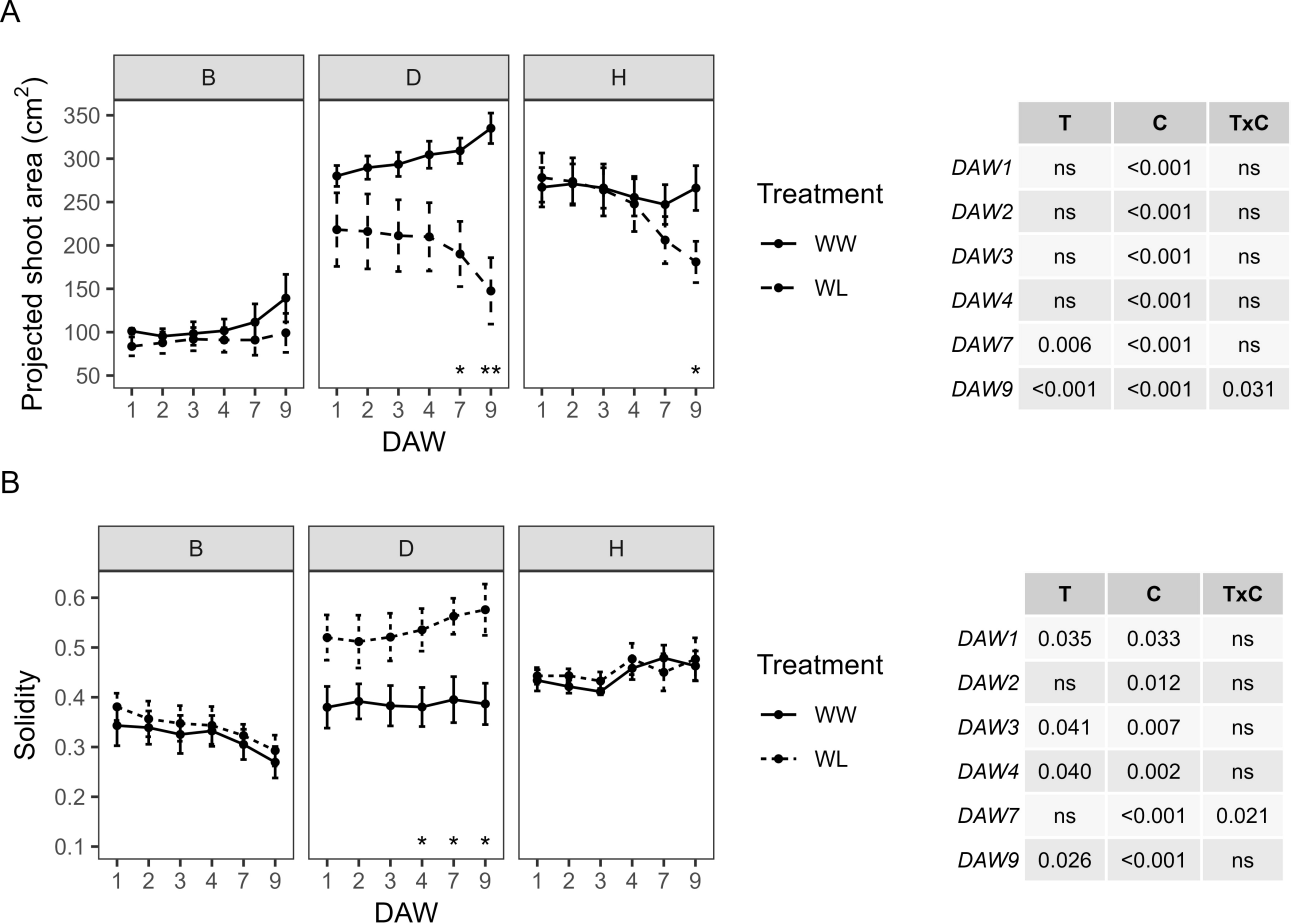
Morphometric parameters derived from the image-based analysis of well-watered (WW) and waterlogged (WL) kiwifruit vines per rootstock (B, D, H): **(A)** projected shoot area (PSA) and **(B)** solidity (S). Two-way ANOVA *p*-values are shown in the tables for both PSA and S and for each DAW. Asterisks denote significant differences according to one-way ANOVA between WW and WL for each parameter and rootstock. Data shown are means ± standard error of the means (*n* = 5). T, treatment effects; C, cultivar effects; ns, not significant at *p* ≥0.05.

RGB image-based parameters that explain plant morphological and color characteristics, in particular PSA, S, plant color (HUE), and SI, were used to study the response of kiwifruit vines to waterlogging. The WW vines of the B and D rootstocks showed a progressive increase in PSA values over the following days of analysis. PSA differed between cultivars, with D and H showing the highest values approximately 280–335 cm^2^ and 250–270 cm^2^, respectively, and B showing the lowest values approximately 95–140 cm^2^ (**Figure 5A**). A predominant effect of the cultivar was observed for PSA throughout the experiment, with the treatment effect appearing only from DAW 7. PSA started to differ significantly between WW and WL vines of the D and H rootstocks from DAW 7 and DAW 9, respectively, reaching values reduced by 56% and 32% compared to WW vines (**Figure 5A**). Projected shoot area and convex hull area from plant images were used to determine leaf density and spread, expressed by the S indicator. S was similar between WW and WL vines of the B and H rootstocks, with no significant differences along the days of waterlogging stress and values approximately 0.3–0.4 and 0.4–0.5, respectively. Instead, it started to differ significantly between WW and WL vines of the D rootstock from DAW 4, resulting in an increase under waterlogging stress. The predominant effect of cultivar was also reported for S and with a lesser effect of treatment (**Figure 5B**).

**Figure 6:**
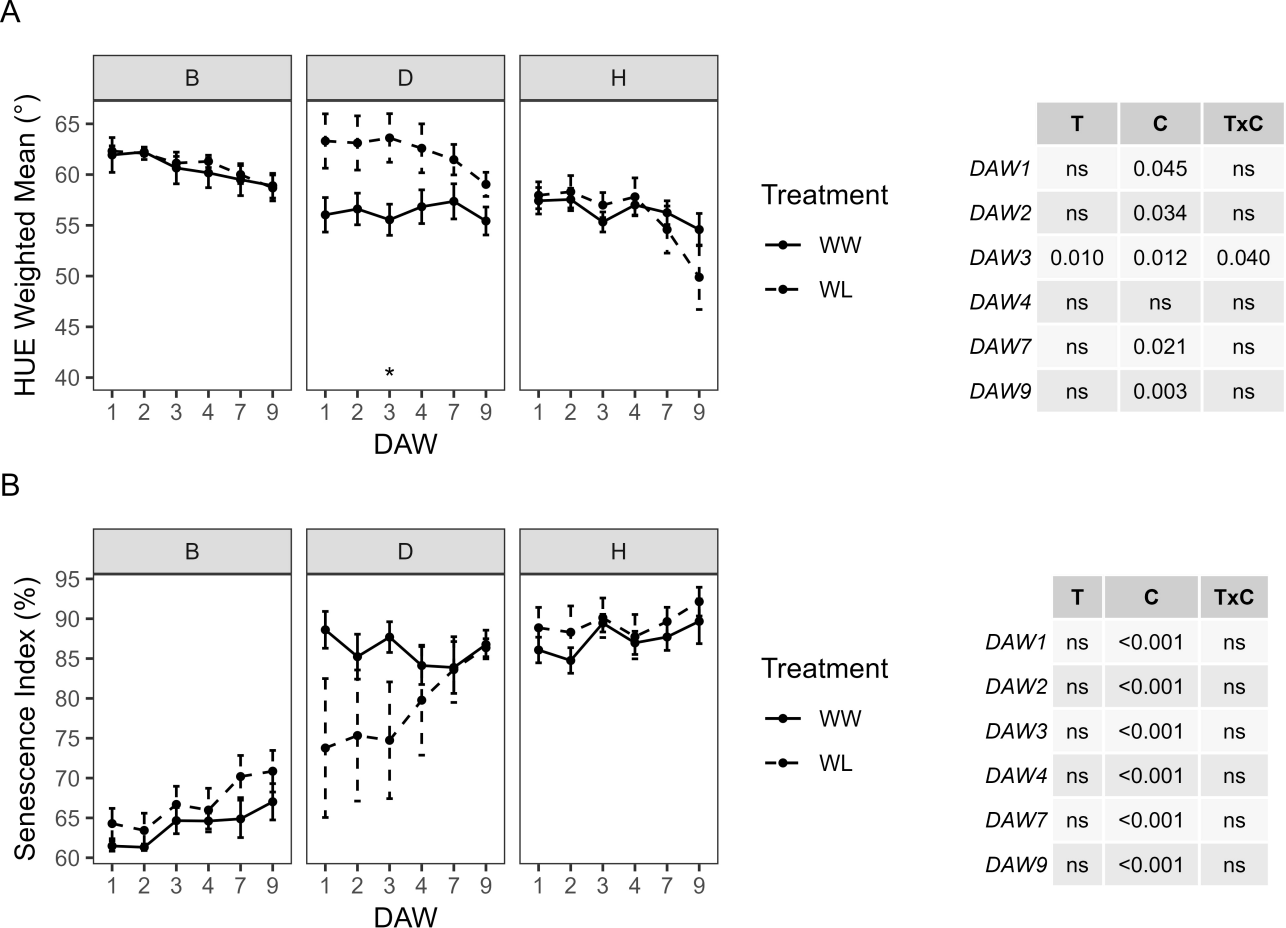
Colorimetric parameters derived from the image-based analysis of well-watered (WW) and waterlogged (WL) kiwifruit vines per rootstock (B, D, H): **(A)** weighted mean value of the hue channel (HUE) and **(B)** senescence index (SI). Two-way ANOVA *p*-values are shown in the tables for both HUE and SI and for each DAW. Asterisks denote significant differences according to one-way ANOVA between WW and WL for each parameter and rootstock. Data shown are means ± standard error of the means (*n* = 5). T, treatment effects; C, cultivar effects; ns, not significant at *p* ≥0.05.

Color changes in kiwifruit vines resulting from the presence of healthy green plant tissue, leaf discoloration, yellowing, or browning of plant tissue were determined by analyzing RGB images with the hue component. During the waterlogging experiment, HUE showed no significant differences between WW and WL vines of each rootstock. However, WL vines of the D and H rootstocks showed a progressive decrease in HUE from DAW 4, whereas those of the B rootstock remained at almost constant values. The WW vines of the D and H rootstocks were similar with values approximately 55°–58°, while those of the B rootstock were slightly higher, above 60° (**Figure 6A**). The proportion between the green and the greener areas was used to determine the SI. No significant differences were found between WW and WL vines of each rootstock during the days of waterlogging stress (**Figure 6B**). The B rootstock had the lowest SI compared to the D and H rootstocks, and the analysis confirmed the prevailing effect of the cultivar for both colorimetric parameters, i.e., HUE and SI, throughout the experiment.

**Figure 7:**
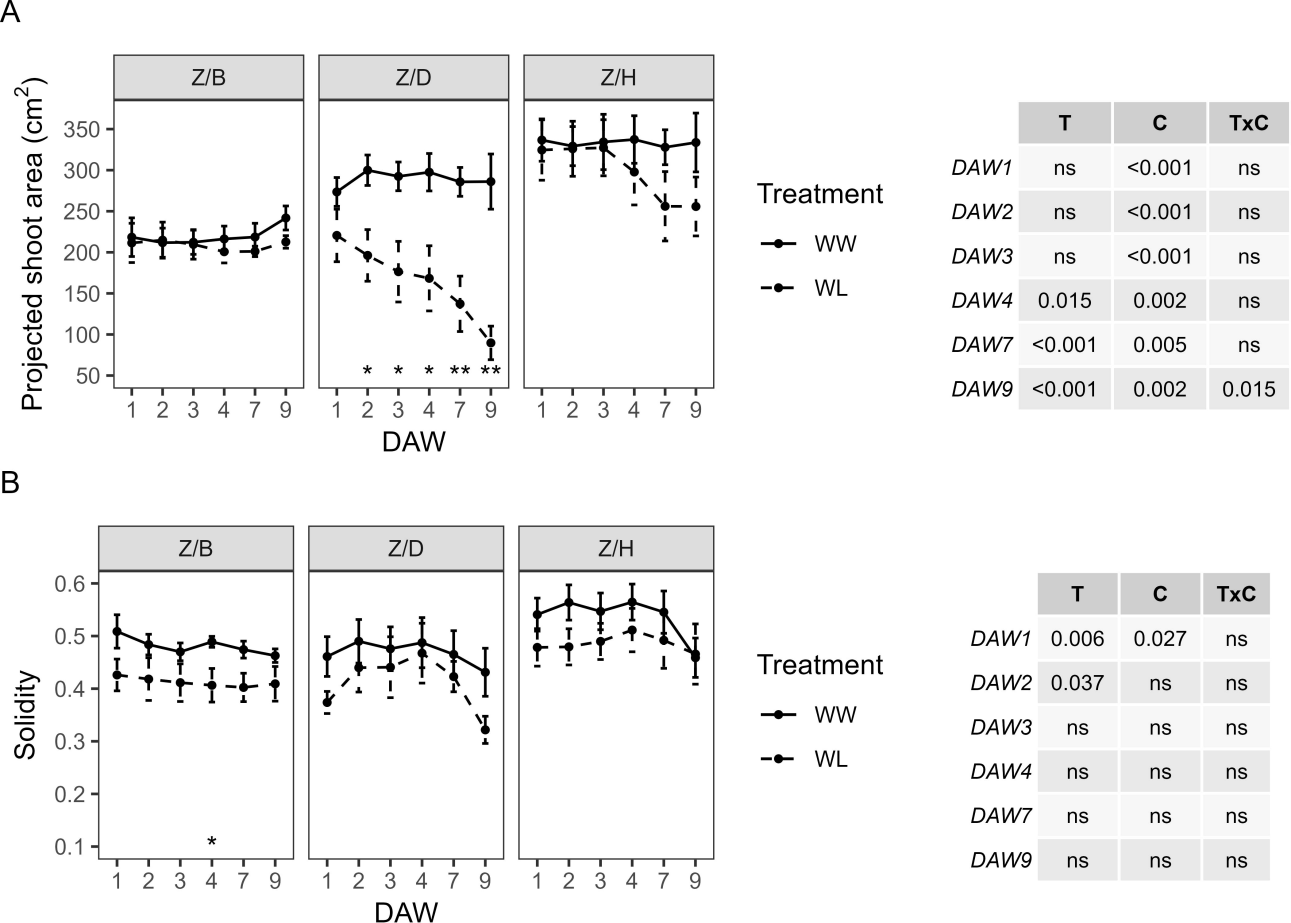
Morphometric parameters derived from the image-based analysis of well-watered (WW) and waterlogged (WL) kiwifruit vines per scion–rootstock combination (Z/B, Z/D, Z/H): **(A)** projected shoot area (PSA) and **(B)** solidity (S). Two-way ANOVA *p*-values are shown in the tables for both PSA and S and for each DAW. Asterisks denote significant differences according to one-way ANOVA between WW and WL for each parameter and scion–rootstock combination. Data shown are means ± standard error of the means (*n* = 5). T, treatment effects; C, cultivar effects; ns, not significant at *p* ≥0.05.

PSA also varied considerably between the scion–rootstock combinations, with scions grafted on the D and H rootstocks showing the highest values approximately 270–300 cm^2^ and 330–340 cm^2^, respectively, and those grafted on the B rootstock showing the lowest values approximately 210–240 cm^2^ (**Figure 7A**). A significant effect of the cultivar on PSA was confirmed, with the treatment effect only appearing from DAW 4 and with consistent differences from DAW 7 (*p* < 0.001). Significant differences in PSA between WW and WL vines of the Z/D combination were found from DAW 2, with a decrease of approximately 70% compared to WW vines at the end of the waterlogging experiment. PSA started to decrease in WL vines of the Z/H combination from DAW 4, but with no significant differences compared to WW vines, and remained almost similar between WW and WL vines of the Z/B combination throughout the experiment (**Figure 7A**). S was similar between WW and WL vines of each scion–rootstock combination, with no significant differences along the days of waterlogging stress and values approximately 0.4–0.5, 0.3–0.5, and 0.45–0.6 for the Z/B, Z/D, and Z/H combinations, respectively (**Figure 7B**).

**Figure 8:**
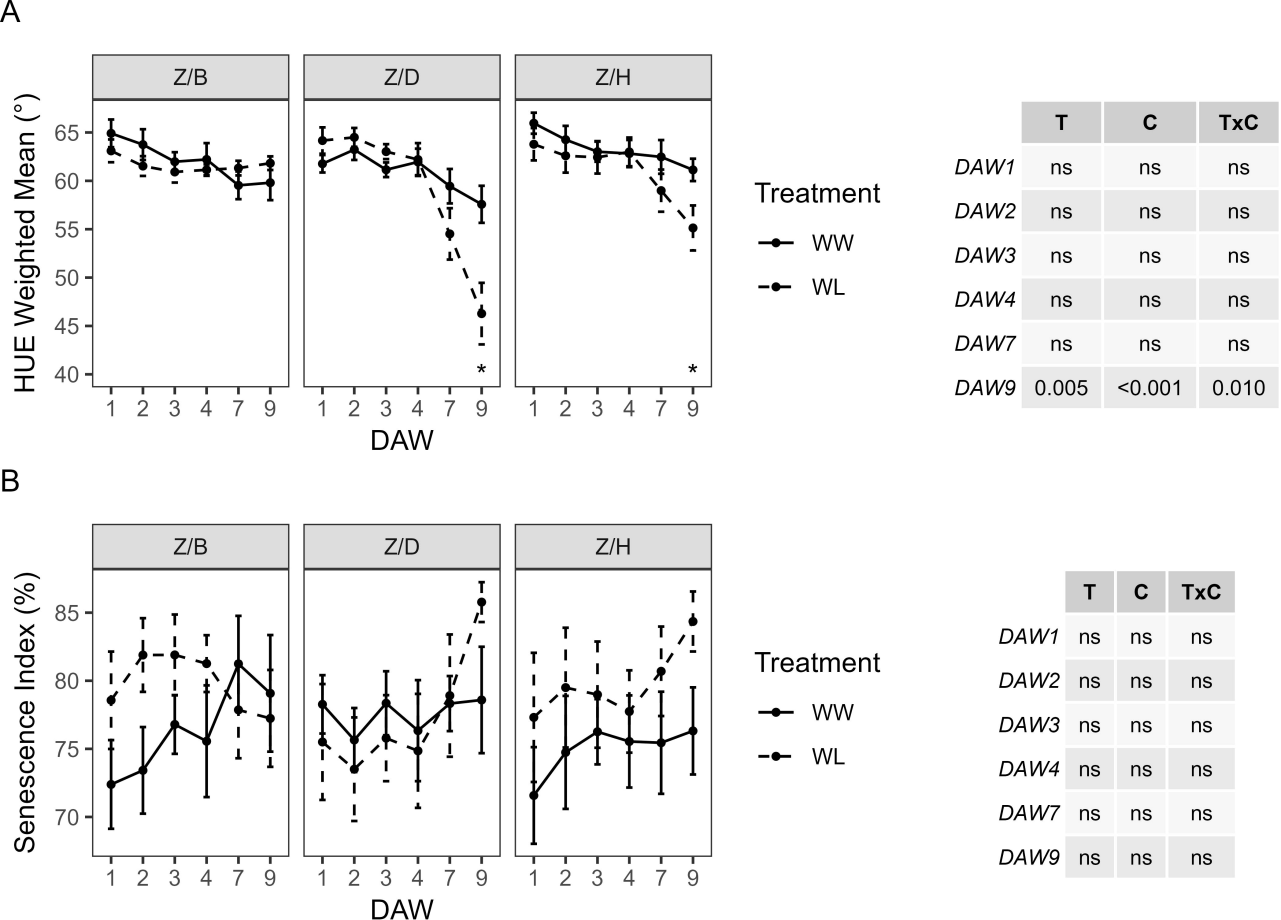
Colorimetric parameters derived from the image-based analysis of well-watered (WW) and waterlogged (WL) kiwifruit vines per scion–rootstock combination (Z/B, Z/D, Z/H): **(A)** weighted mean value of the hue channel (HUE) and **(B)** senescence index (SI). Two-way ANOVA *p*-values are shown in the tables for both HUE and SI and for each DAW. Asterisks denote significant differences according to one-way ANOVA between WW and WL for each parameter and scion–rootstock combination. Data shown are means ± standard error of the means (*n* = 5). T, treatment effects; C, cultivar effects; ns, not significant at *p* ≥0.05.

The HUE of WW vines was almost similar between the different scion–rootstock combinations, showing a slight decrease from DAW 4 in all combinations. No significant differences in HUE were found between WW and WL vines of the Z/B combination. HUE in WL vines of the Z/D and Z/H combinations started to decrease from DAW 4 and differed significantly from WW vines at DAW 9, where both treatment and cultivar had a significant effect (**Figure 8A**). No significant effects of cultivar and treatment on SI were found by two-way ANOVA analysis, and no differences were found between WW and WL vines of each scion–rootstock combination during the days of waterlogging stress (**Figure 8B**).

### 3.3 Near-infrared imaging

**Figure 9:**
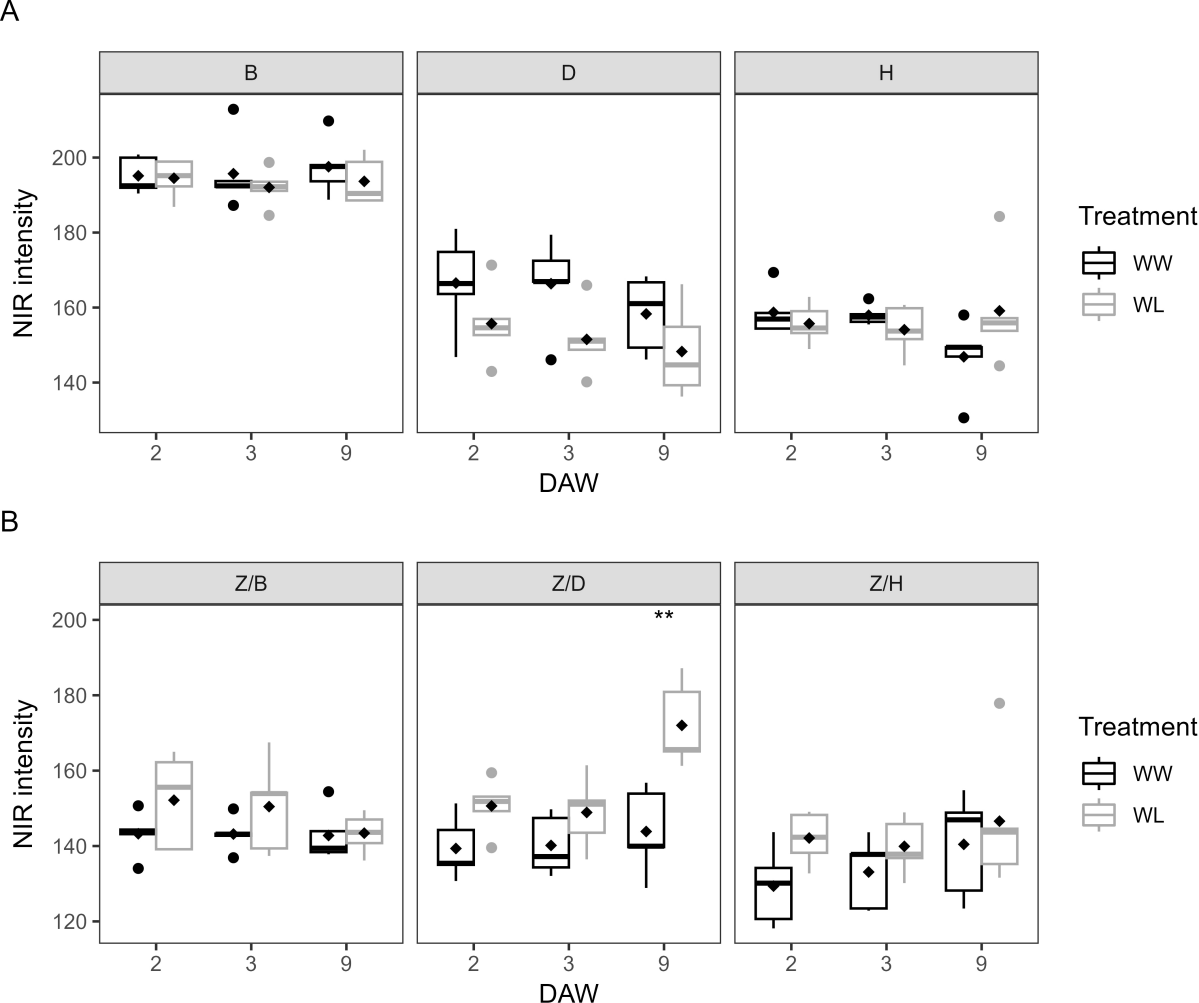
NIR intensities of well-watered (WW) and waterlogged (WL) kiwifruit vines: **(A)** per rootstock (B, D, H) and **(B)** per scion–rootstock combination (Z/B, Z/D, Z/H). Asterisks denote significant differences according to one-way ANOVA between WW and WL vines at each DAW. Black diamond symbols represent mean values (*n* = 5).

NIR imaging analysis is a common tool used to assess the water content of plants. During the waterlogging days, NIR intensity was similar between WW and WL vines of each rootstock, with no significant differences (**Figure 9A**). However, regardless of the irrigation treatment applied, the B rootstock showed the highest NIR intensity, with values close to 200, and thus a lower water content in plant tissues compared to the D and H rootstocks, with values between 150 and 170 (**Figure 9A**). In the scion–rootstock combinations, NIR intensity reached higher values in all WL vines compared to WW vines (**Figure 9B**). During the last day of waterlogging stress (DAW 9), NIR intensity differed significantly between WW and WL vines of the Z/D combination, indicating a reduced water content of the vines achieved after the application of 9 days of waterlogging stress (**Figure 9B**). NIR intensity was then a good indicator of plant stress response, in accordance with the changes in morphometric and colorimetric parameters in response to waterlogging in kiwifruit.

## 4 Discussion

The results of the present study outlined the differential response of kiwifruit rootstocks and grafting combinations to waterlogging stress. Physiological and phenotypic traits of the vines were combined to increase knowledge of vine physiological behavior and RGB and NIR image-based indicators for assessing waterlogging tolerance/susceptibility in kiwifruit genotypes.

### 4.1 Physiological waterlogging response

Stomatal closure is widely recognized as one of the most rapid responses of many species to waterlogging stress (Kozlowski, 1997; Parent et al., 2008; Xu et al., 2022; Topali et al., 2024). Stomata are responsible for regulating A and E by opening and closing (Kozlowski, 1997), and their rapid response is correlated with an early reduction in A since the beginning of waterlogging (Xu et al., 2022). Results showed that gs and therefore E were affected by waterlogging from the first days of the experiment in both D and H rootstocks, but not in the B rootstock. Stomatal closure shortly after waterlogging and a decrease in net photosynthesis have been previously reported in kiwifruit (Li et al., 2020; Li Z. et al., 2021) and in other fruit tree species such as *Prunus* spp (Domingo et al., 2002; Pimentel et al., 2014; Zhang et al., 2023). This tendency was confirmed as the values of A generally decreased with those of gs, specifically from DAW 4 in the D and H rootstocks and from DAW 2 in the Z/D and Z/H combinations.

A more rapid decrease in gs was induced under waterlogging in all grafting combinations and slightly delayed in the Z/B combination.

This suggests that the B rootstock may be more tolerant to waterlogging stress than the D and H rootstocks and that the rootstock may influence the response of the grafted cultivar when exposed to waterlogging stress. Rootstocks with different waterlogging tolerance were found to influence the photosynthetic capacity of the scion, maintaining it for a longer time under waterlogging stress in peach (Zhang et al., 2023), and to improve the physiological, biochemical, and molecular responses of the scion in kiwifruit (Bai et al., 2022). This influence, which is only reflected in the Z/B combination, may be of great importance in increasing the potential tolerance of kiwifruit to excess water in poorly structured soils and should therefore be taken into account in the genotypic selection of the rootstock. However, the waterlogging tolerance of the B rootstock is not fully reflected in the grafting combination.

In particular, gs followed by a rapid decrease in A was previously observed in 3-month-old plants of ‘Hayward’ under waterlogging stress, suggesting a high sensitivity of this rootstock to waterlogging conditions (Li Z. et al., 2021). Although no information is available specifically for the D rootstock, the data analyzed suggest that the D and H rootstocks behave similarly under waterlogging stress, identifying them as waterlogging-sensitive rootstocks. Both rootstocks belong to *A. chinensis* var. *deliciosa* which has been found to have a low tolerance to waterlogging (Youn-Seop et al., 2008; Bai et al., 2019; Kataoka et al., 2021; Beppu et al., 2022). The observed physiological behavior of the B rootstock, which was not affected by the 9-day waterlogging treatment, was consistent with previous studies showing that A, gs, and E in *A. macrosperma* were maintained at relatively high levels even under waterlogging stress (Kataoka et al., 2021; Beppu et al., 2022), together with high root activity (Bai et al., 2019). Furthermore, Kataoka et al. (2021) found that grafting ‘Hayward’ onto *A. macrosperma* rootstock allowed greater tolerance to be expressed in the grafted kiwifruit scion. This is consistent with the results of the present study, where the physiological responses of the scion grafted on the B rootstock to waterlogging stress were delayed in time compared to other grafting combinations.

Maintaining an efficient and effective photosynthetic activity and undamaged photosynthetic apparatus is extremely important for plant growth and development (Zhang et al., 2023). Photosynthetic gas exchange parameters can be used to assess plant tolerance to waterlogging. Waterlogging stress can induce stomatal closure, which limits leaf gas exchange and leads to reduced CO_2_ absorption, thereby affecting photosynthetic rate (Pan et al., 2021). The initial reduction in A is reported to be highly correlated with stomatal closure, but after prolonged periods of waterlogging, the rate of A also decreases due to the inhibitory effects on the photosynthetic process (Kozlowski, 1997). By analyzing the different trends in Ci values, it is possible to detect the presence of mechanisms other than gs (non-stomatal factors) that influence the assimilation process and thus photosynthetic rates. Lower Ci values in the WL vines of the Z/D and Z/H combinations supported that the leaves were CO_2_-limited due to lower gs. Conversely, Pimentel et al. (2014) found increased Ci values in waterlogged plants of *Prunus* species, suggesting that the leaves were not CO_2_-limited and that lower A was determined by non-stomatal factors. Similar results were instead found by Baldi et al. (2024) in ‘Hayward’ kiwifruit, where high reductions in A were observed in plants exposed to waterlogging, mainly associated with significantly lower gs, resulting in lower Ci values. The higher Ci value found in WL vines of the Z/D combination at DAW 4 indicated the occurrence of non-stomatal factors, identifying a later stage of waterlogging stress when photosynthetic metabolism was almost irreversibly damaged, as indicated by very low A. Instead, WL vines of the Z/H combination maintained a lower Ci also at DAW 4, confirming the effect of lower gs on the assimilation process and thus no damage to the photosynthetic apparatus already at this time. On this basis, a slightly lower sensitivity of the Z/H combination to waterlogging stress compared to the Z/D combination is suggested. The results concerning WUEi are consistent with Xu et al. (2022), who showed that some peach rootstocks maintained high levels of WUE before gradually declining until the end of the stress and that very low values may indicate irreversible damage to photosynthetic properties. Lower WUEi values in WL vines of the Z/D combination at DAW 4 may indicate the beginning of a damage process to the photosynthetic system. Mature leaves of kiwifruit vines are characterized by large dimensions and an orbicular shape in the D and H rootstocks and in the grafted yellow-fleshed cultivar, features that contribute to increased leaf temperatures (Smith et al., 1989). Significant differences in T_leaf_ between WW and WL vines can be attributed to significant reductions in gs and E, which determine the attainment of lethal temperatures in parts of the leaves that can be irreversibly damaged (Smith et al., 1989). In the present study, the physiological responses to waterlogging stress varied between the three kiwifruit rootstocks and grafting combinations. Early reductions in leaf gas exchange under waterlogging stress were observed in the D and H rootstocks compared to the B rootstock, which was not affected throughout the experiment. In particular, the reductions were more pronounced in the grafting combinations where the influence of the rootstock on the scion was evident.

### 4.2 Image-based parameters to phenotype for waterlogging stress

Many studies have used image analysis to investigate the responses of plants to various abiotic stresses (e.g., drought, heat, and salt stress) (Briglia et al., 2019, 2020; Kim et al., 2020; Carvalho et al., 2021). Non-destructive and non-invasive phenotyping assessment of waterlogging responses using a high-throughput plant phenotyping platform in fruit tree crops has been poorly addressed, and the results obtained in the present study represent a novelty for the kiwifruit crop.

The detrimental effect of waterlogging stress on plant growth and development is well known (Manghwar et al., 2024), and plant biomass is an important trait assessed to investigate the waterlogging response of plants (Langan et al., 2022). Among the morphological parameters that can be assessed non-destructively by image analysis, PSA and S may be relevant indicators to evaluate kiwifruit vine development and changes in leaf density and spread as influenced by waterlogging. Traditional phenotypic observations can be influenced by cultivar-specific traits such as leaf surface area and maturity (Zhang et al., 2023), which in turn affect their response to waterlogging stress. The waterlogging tolerance or sensitivity of rootstocks can directly influence scion growth and development in kiwifruit (Bai et al., 2019).

Significant differences in PSA and S between rootstocks can be attributed to differences in leaf number, size, shape, and anatomy, particularly distinguishing the B rootstock from the D and H rootstocks. The reductions in PSA that occurred in the D and H rootstocks under waterlogging showed an effect of waterlogging stress on total vine area. Previous phenotyping studies have used PSA or similar indices of plant biomass in several crops and found similar deleterious effects of drought stress on plant aerial mass (Danzi et al., 2019; Briglia et al., 2020; Kim et al., 2020; Genangeli et al., 2023; Petrozza et al., 2023), reinforcing the issue that waterlogging stress affects leaves in a similar way to drought stress (Topali et al., 2024). The PSA evaluated in the grafting combinations confirmed the results found in the rootstocks, with a more pronounced effect of waterlogging stress on the aerial mass of the vines of the Z/D and Z/H combinations.

In the present study, the most sensitive D rootstock showed a higher S under waterlogging stress, as determined by the lower convex hull area values of WL vines compared to WW vines, although waterlogging induced a decrease in PSA. Higher values of S were previously observed in a drought experiment carried out on tomato plants, where drought-stressed plants lost turgor and reduced their convex hull area, resulting in increased S (Janni et al., 2019). However, reduced S has also been suggested as a consequence of water stress and could be determined by decreasing the plant area pixels while keeping the convex hull area constant (Petrozza et al., 2023). In the case of the grafting combinations, S values were lower in the WL vines compared to the WW vines, with a decreasing trend with prolonged waterlogging exposure in the Z/D and Z/H combinations, indicating a gradual wilting of the leaves, which is consistent with considering lower S values as a consequence of stress. Petrozza et al. (2023) used S as an index of the differential response of tomato plants to drought stress and reported lower S in sensitive plants under drought conditions, which was explained as a consequence of the reduced plant pixel area due to greater wilting of drought-sensitive plants. S is then a relative index that varies between species and even cultivars according to the specific behavior of plants to a given stress.

Waterlogging stress often causes leaf chlorosis, wilting, and yellowing (Langan et al., 2022), reducing green leaf area (Ren et al., 2023). The leaves turn yellow and develop necrosis as a result of prolonged exposure to waterlogging at the roots, leading to abscission (Topali et al., 2024). Furthermore, changes in the balance of leaf catabolism and anabolism processes during waterlogging stress have been reported to cause a decrease in leaf chlorophyll content (Zhang et al., 2023), which could contribute to reduced photosynthesis and leaf color change, which gradually turn from green to yellow with prolonged exposure to waterlogging. The RGB images have great potential not only for morphological studies, including parameters such as PSA and S, but also for leaf color estimation. In particular, leaf chlorosis, which is an indication of the chlorophyll degradation and contributes to a decrease in A, can be detected by measuring the number of pixels in the green and yellow regions of the hue channels. Significant differences in HUE in WL vines of the Z/D and Z/H combinations may indicate the beginning of a process of leaf chlorosis induced by waterlogging. Assessment of leaf color, degree of yellowing, or loss of greenness has been reported as a promising tool to detect the occurrence of drought stress, as an increasing fraction of the yellow color class was associated with increasing drought in a grapevine experiment (Briglia et al., 2019). The SI was used to indicate the senescence status of the vines. No significant differences in SI were found between the irrigation treatments, although an increasing trend was observed in WL vines of all rootstocks and Z/D and Z/H combinations.

While the effects of waterlogging were registered almost immediately in the RGB morphometric parameters, the color changes appeared with a delay and with greater variability between the vines, in agreement with other studies (Janni et al., 2019; Genangeli et al., 2023). The results indicate that the RGB image-based parameters that most accurately represented the differential response of kiwifruit rootstocks and grafting combinations to waterlogging were plant PSA and HUE color class. These parameters can be effective indicators for screening for more tolerant kiwifruit genotypes using non-destructive and non-invasive plant phenotyping.

The water content of plant tissues is known to influence spectral reflectance, and therefore, non-destructive techniques such as those based on NIR wavelengths are useful for assessing plant water status (Kim et al., 2020; Danzi et al., 2022). NIR intensity values showed an increasing trend (lower water content) in all WL vines of the grafting combinations, suggesting that waterlogging affected the water status of the vines as detected by NIR images. The water content of plant tissues was then reduced under waterlogging stress, which has been reported as an effect of waterlogging on plants (Sathi et al., 2022). The occurrence of partial leaf wilting and increased leaf surface necrosis area contributed to the reduction in water content of tissues that began to dry out.

Physiological measurements and RGB analysis identified the Z/D combination as the most sensitive to waterlogging stress, followed by the Z/H combination. Furthermore, 4 days of waterlogging stress can be identified as the time when a more severe stress occurred for the Z/D and Z/H combinations, as all image-based parameters began to change from the control values. Although the Z/B combination showed a delayed decrease in physiological activity compared to the other combinations, it did not show any changes in morphometric and colorimetric parameters under waterlogging stress. RGB image-based results confirmed that the B rootstock enhanced the tolerance of the scion to waterlogging stress.

## 5 Conclusions

Waterlogging and root hypoxia conditions are predicted to become more severe due to anomalous climatic conditions, and represent a serious threat that is increasingly affecting kiwifruit cultivation. Novel results are presented on the assessment of the waterlogging response of different kiwifruit rootstocks and grafting combinations by combining traditional physiological measurements with high throughput phenotypic analysis. Physiological performances and image-based parameters were negatively affected by soil waterlogging, but to different extents in the kiwifruit rootstocks and grafting combinations studied. D and H rootstocks showed an early reduction in stomatal conductance and photosynthetic rates, and an increase in leaf temperature already during the first days of waterlogging stress, confirming that these parameters are effective indicators of altered behavior caused by waterlogging, as by other abiotic stresses RGB and NIR image analysis, through the evaluation of parameters such as projected shoot area, leaf color, and plant tissue water content, supported the waterlogging sensitivity of the D and H rootstocks and their combinations and provided promising indicators to be used in the evaluation of the waterlogging response of kiwifruit vines. Instead, B rootstock was unaffected by 9 days of waterlogging stress in both physiological and phenotyping parameters. Waterlogging effects were more pronounced in the yellow-fleshed kiwifruit cultivar grafted on D and H rootstocks than in the cultivar grafted on B rootstock, suggesting an influence of the rootstock on the scion. However, stomatal conductance also decreased in the Z/B combination, suggesting that the B rootstock is more tolerant to waterlogging, but not able to allow the grafted cultivar to cope with prolonged and adverse soil conditions. More efforts should be made to adopt proper orchard management and to promote breeding research for the selection of well-adapted plant material (rootstocks and cultivars). The present study reveals the suitability of RGB morphometric and colorimetric parameters analyzed on kiwifruit vines under waterlogging stress as promising indicators. In conclusion, physiological and phenotyping assessment can be an effective methodology for screening for more waterlogging tolerant genotypes for the kiwifruit crop.

## Data Availability

The raw data supporting the conclusions of this article will be made available by the authors, without undue reservation.

## References

[B1] Abid M. Zhang Y. J. Li Z. Bai D. F. Zhong Y. P. Fang J. B. (2020). Effect of salt stress on growth, physiological and biochemical characters of four kiwifruit genotypes. Scientia Hortic. 271, 109473. doi: 10.1016/j.scienta.2020.109473

[B2] Bai D. Li Z. Gu S. Li Q. Sun L. Qi X.. (2022). Effects of kiwifruit rootstocks with opposite tolerance on physiological responses of grafting combinations under waterlogging stress. Plants 11, 2098. doi: 10.3390/plants11162098 36015401 PMC9416424

[B3] Bai D. F. Li Z. Qi X. J. Chen J. Y. Gu H. Huang W. Q.. (2019). Physiological responses and tolerance evaluation of four species of Actinidia to waterlogging stress. J. Fruit Sci. 36, 163–173. doi: 10.13925/j.cnki.gsxb.20180304

[B4] Baldi E. Pastore C. Chiarelli G. Quartieri M. Spinelli F. Toselli M. (2024). Molecular Responses to Drought and Waterlogging Stresses of Kiwifruit (Actinidia chinensis var. deliciosa) Potted Vines. Horticulturae 10, 834. doi: 10.3390/horticulturae10080834

[B5] Bardi L. (2020). Early kiwifruit decline: A soil-borne disease syndrome or a climate change effect on plant–soil relations? Front. Agron. 2. doi: 10.3389/fagro.2020.00003

[B6] Bardi L. Nari L. Morone C. Solomita M. Mandalà C. Faga M. G.. (2022). Kiwifruit adaptation to rising vapor pressure deficit increases the risk of kiwifruit decline syndrome occurrence. Horticulturae, 8 (10), 906. doi: 10.3390/horticulturae8100906

[B7] Bellasio C. (2023). The slope of assimilation rate against stomatal conductance should not be used as a measure of water use efficiency or stomatal control over assimilation. Photosyn. Res. 158, 195–199. doi: 10.1007/s11120-023-01054-6 PMC10695868 37902923

[B8] Beppu K. Ogihara Y. Ohtani M. Kataoka I. (2022). Comparison of waterlogging tolerance between *Actinidia macrosperma* and *Actinidia deliciosa* . Acta Hortic. 1332, 219–226. doi: 10.17660/ActaHortic.2022.1332.29

[B9] Briglia N. Montanaro G. Petrozza A. Summerer S. Cellini F. Nuzzo V. (2019). Drought phenotyping in Vitis vinifera using RGB and NIR imaging. Scientia Hortic. 256, 108555. doi: 10.1016/j.scienta.2019.108555

[B10] Briglia N. Williams K. Wu D. Li Y. Tao S. Corke F.. (2020). Image-based assessment of drought response in grapevines. Front. Plant Sci. 11. doi: 10.3389/fpls.2020.00595 PMC7242646 32499808

[B11] Burdon J. Pidakala P. Martin P. McAtee P. A. Boldingh H. L. Hall A.. (2014). Postharvest performance of the yellow-fleshed “Hort16A” kiwifruit in relation to fruit maturation. Postharvest Biol. Technol. 92, 98–106. doi: 10.1016/j.postharvbio.2014.01.004

[B12] Carvalho L. C. Gonçalves E. F. Marques da Silva J. Costa J. M. (2021). Potential phenotyping methodologies to assess inter-and intravarietal variability and to select grapevine genotypes tolerant to abiotic stress. Front. Plant Sci. 12. doi: 10.3389/fpls.2021.718202 PMC8575754 34764964

[B13] Clearwater M. J. Lowe R. G. Hofstee B. J. Barclay C. Mandemaker A. J. Blattmann P. (2004). Hydraulic conductance and rootstock effects in grafted vines of kiwifruit. J. Exp. Bot. 55, 1371–1382. doi: 10.1093/jxb/erh137 15133051

[B14] Cradock-Henry N. A. (2017). New Zealand kiwifruit growers’ vulnerability to climate and other stressors. Reg. Environ. Change 17, 245–259. doi: 10.1007/s10113-016-1000-9

[B15] Danzi D. Briglia N. Petrozza A. Summerer S. Povero G. Stivaletta A.. (2019). Can high throughput phenotyping help food security in the mediterranean area? Front. Plant Sci. 10, 15. doi: 10.3389/fpls.2019.00015 30740116 PMC6355677

[B16] Danzi D. De Paola D. Petrozza A. Summerer S. Cellini F. Pignone D.. (2022). The use of near-infrared imaging (NIR) as a fast non-destructive screening tool to identify drought-tolerant wheat genotypes. Agriculture 12, 537. doi: 10.3390/agriculture12040537

[B17] Di Biase R. Calabritto M. Sofo A. Reyes F. Mininni A. N. Mastroleo M.. (2023). Assessment of kiwifruit physiological decline: irrigation and soil management strategy to recover from waterlogging. Acta Hortic. 1373, 11–18. doi: 10.17660/ActaHortic.2023.1373.3

[B18] Domingo R. Pérez-Pastor A. Ruiz-Sánchez C. (2002). Physiological responses of apricot plants grafted on two different rootstocks to flooding conditions. J. Plant Physiol. 159, 725–732. doi: 10.1078/0176-1617-0670

[B19] Donati I. Cellini A. Sangiorgio D. Caldera E. Sorrenti G. Spinelli F. (2020). Pathogens associated to kiwifruit vine decline in Italy. Agriculture 10, 119. doi: 10.3390/agriculture10040119

[B20] Fahlgren N. Feldman M. Gehan M. A. Wilson M. S. Shyu C. Bryant D. W.. (2015). A versatile phenotyping system and analytics platform reveals diverse temporal responses to water availability in Setaria. Mol. Plant 8, 1520–1535. doi: 10.1016/j.molp.2015.06.005 26099924

[B21] FAO (2022). FAOSTAT—Food and Agriculture Organization of the United Nations (Rome, Italy). Available online at: https://www.fao.org/faostat/en/home (Accessed April 2024).

[B22] Friend A. P. Palmer J. W. Seymour S. M. Diack R. N. (2014). Potential of clonal rootstocks for devigoration and enhanced fruit characteristics in kiwifruit orchards. Acta Hortic. 1058, 429–434. doi: 10.17660/ActaHortic.2014.1058.52

[B23] Fullana-Pericàs M. Conesa M. Pérez-Alfocea F. Galmés J. (2020). The influence of grafting on crops’ photosynthetic performance. Plant Sci. 295, 110250. doi: 10.1016/j.plantsci.2019.110250 32534620

[B24] Genangeli A. Avola G. Bindi M. Cantini C. Cellini F. Grillo S.. (2023). Low-cost hyperspectral imaging to detect drought stress in high-throughput phenotyping. Plants 12, 1730. doi: 10.3390/plants12081730 37111953 PMC10143644

[B25] Golzarian M. R. Frick R. A. Rajendran K. Berger B. Roy S. Tester M.. (2011). Accurate inference of shoot biomass from high-throughput images of cereal plants. Plant Methods 7, 1–11. doi: 10.1186/1746-4811-7-2 21284859 PMC3042986

[B26] Holzapfel E. A. Merino R. Mariño M. A. Matta R. (2000). Water production functions in kiwi. Irrig. Sci. 19, 73–79. doi: 10.1007/s002710050003

[B27] Janni M. Coppede N. Bettelli M. Briglia N. Petrozza A. Summerer S.. (2019). *In vivo* phenotyping for the early detection of drought stress in tomato. Plant Phenom. 1–10. doi: 10.34133/2019/6168209 PMC7706337 33313533

[B28] Kataoka I. Yamada T. Fukuda T. Ohtani M. Katsuhiko S. Beppu K. (2021). Waterlogging tolerance of Actinidia macrosperma and its application to kiwifruit by grafting. Hortic. Res. 20, 265–271. doi: 10.2503/hrj.20.265

[B29] Kim S. L. Kim N. Lee H. Lee E. Cheon K. S. Kim M.. (2020). High-throughput phenotyping platform for analyzing drought tolerance in rice. Planta 252, 38. doi: 10.1007/s00425-020-03436-9 32779032 PMC7417419

[B30] Kozlowski T. T. (1997). Responses of woody plants to flooding and salinity. Tree Physiol. 17, 490–490. doi: 10.1093/treephys/17.7.490

[B31] Langan P. Bernád V. Walsh J. Henchy J. Khodaeiaminjan M. Mangina E.. (2022). Phenotyping for waterlogging tolerance in crops: current trends and future prospects. J. Exp. Bot. 73, 5149–5169. doi: 10.1093/jxb/erac243 35642593 PMC9440438

[B32] Li Z. Bai D. Zhong Y. Abid M. Qi X. Hu C.. (2021). Physiological responses of two contrasting kiwifruit (*Actinidia* spp.) rootstocks against waterlogging stress. Plants, 10(12), 2586. doi: 10.3390/plants10122586 PMC8707060 34961057

[B33] Li D. Han F. Liu X. Lv H. Li L. Tian H.. (2021). Localized graft incompatibility in kiwifruit: Analysis of homografts and heterografts with different rootstock & scion combinations. Sci. Hortic. 283, 110080. doi: 10.1016/j.scienta.2021.110080

[B34] Li Z. Zhong Y. Bai D. Lin M. Qi X. Fang J. (2020). Comparative analysis of physiological traits of three Actinidia valvata Dunn genotypes during waterlogging and post-waterlogging recovery. Hortic. Environ. Biotechnol. 61, 825–836. doi: 10.1007/s13580-020-00276-0

[B35] Manghwar H. Hussain A. Alam I. Khoso M. A. Ali Q. Liu F. (2024). Waterlogging stress in plants: Unraveling the mechanisms and impacts on growth, development, and productivity. Environ. Exp. Bot. 224, 105824. doi: 10.1016/j.envexpbot.2024.105824

[B36] Mian G. Cipriani G. Saro S. Martini M. Ermacora P. (2022b). Potential of different actinidia genotypes as resistant rootstocks for preventing kiwifruit vine decline syndrome. Horticulturae, 8 (7), 627. doi: 10.3390/horticulturae8070627

[B37] Mian G. Iseppi L. Traversari G. Ermacora P. Cipriani G. Nassivera F. (2022a). Consumers perceptions and motivations in the choice of kiwifruits: A study-case in Italy, north-east. Qual. - Access to Success 23, 163–175. doi: 10.47750/QAS/23.188.23

[B38] Pan J. Sharif R. Xu X. Chen X. (2021). Mechanisms of waterlogging tolerance in plants: Research progress and prospects. Front. Plant Sci. 11. doi: 10.3389/fpls.2020.627331 PMC7902513 33643336

[B39] Parent C. Capelli N. Berger A. Crèvecoeur M. Dat J. F. (2008). An overview of plant responses to soil waterlogging. Plant Stress 2, 20–27.

[B40] Petrozza A. Santaniello A. Summerer S. Di Tommaso G. Di Tommaso D. Paparelli E.. (2014). Physiological responses to Megafol^®^ treatments in tomato plants under drought stress: A phenomic and molecular approach. Scientia Hortic. 174, 185–192. doi: 10.1016/j.scienta.2014.05.023

[B41] Petrozza A. Summerer S. Melfi D. Mango T. Vurro F. Bettelli M.. (2023). A Lycopene ϵ-cyclase TILLING allele enhances lycopene and carotenoid content in fruit and improves drought stress tolerance in tomato plants. Genes 14, 1284. doi: 10.3390/genes14061284 37372464 PMC10298496

[B42] Pimentel P. Almada R. D. Salvatierra A. Toro G. Arismendi M. J. Pino M. T.. (2014). Physiological and morphological responses of Prunus species with different degree of tolerance to long-term root hypoxia. Scientia Hortic. 180, 14–23. doi: 10.1016/j.scienta.2014.09.055

[B43] Prencipe S. Schiavon G. Rosati M. Nari L. Schena L. Spadaro D. (2023). Characterization of phytopythium species involved in the establishment and development of kiwifruit vine decline syndrome. Microorganisms, 11 (1), 216. doi: 10.3390/microorganisms11010216 PMC9862930 36677508

[B44] Reid J. B. Tate K. G. Brown N. S. Cheah L. H. (1991). Effects of flooding and alluvium deposition on kiwifruit (Actinidia deliciosa) 1. early vine decline. N Z J. Crop Hortic. Sci. 19, 247–257. doi: 10.1080/01140671.1991.10421808

[B45] Ren B. Yu W. Liu P. Zhao B. Zhang J. (2023). Responses of photosynthetic characteristics and leaf senescence in summer maize to simultaneous stresses of waterlogging and shading. Crop J. 11, 269–277. doi: 10.1371/journal.pone.0161424

[B46] Sairam R. K. Kumutha D. Ezhilmathi K. Deshmukh P. S. Srivastava G. C. (2008). Physiology and biochemistry of waterlogging tolerance in plants. Biol. Plant. 52, 401–412. doi: 10.1007/s10535-008-0084-6

[B47] Sathi K. S. Masud A. A. C. Falguni M. R. Ahmed N. Rahman K. Hasanuzzaman M. (2022). Screening of soybean genotypes for waterlogging stress tolerance and understanding the physiological mechanisms. Adv. Agric. 2022, 5544665. doi: 10.1155/2022/5544665

[B48] Savé R. Serrano L. (1986). Some physiological and growth responses of kiwi fruit (Actinidia chinensis) to flooding. Physiol. Plant. 66, 75–78. doi: 10.1111/j.1399-3054.1986.tb01236.x

[B49] Savian F. Ginaldi F. Musetti R. Sandrin N. Tarquini G. Pagliari L.. (2020). Studies on the aetiology of kiwifruit decline: interaction between soil-borne pathogens and waterlogging. Plant Soil 456, 113–128. doi: 10.1007/s11104-020-04671-5

[B50] Smith B. G. Buwalda J. G. A Green T. G. J Clark N. C. (1989). Effect of oxygen supply and temperature at the root on the physiology of kiwifruit vines. New Phytol. 133, 431–437. doi: 10.1111/j.1469-8137.1989.tb00354.x

[B51] Smith G. S. Judd M. J. Miller S. A. Buwalda J. G. (1990). Recovery of kiwifruit vines from transient waterlogging of the root system. New Phytol. 115, 325–333. doi: 10.1111/j.1469-8137.1990.tb00459.x 33873955

[B52] Sofo A. Dichio B. Elshafie H. S. Camele I. Calabritto M. Tomasi I.. (2024). Enhancing soil properties through sustainable agronomic practices reduced the occurrence of kiwifruit vine decline syndrome. Soil Use Manage. 40, e13052. doi: 10.1111/sum.13052

[B53] Sofo A. Mininni A. N. Dichio B. Mastroleo M. Xylogiannis E. (2022). Physical structure and chemical quality of waterlogged soils in an Italian kiwifruit orchard. Acta Hortic. 1332, 195–202. doi: 10.17660/ActaHortic.2022.1332.26

[B54] Spigaglia P. Barbanti F. Marocchi F. Mastroleo M. Baretta M. Ferrante P.. (2020). *Clostridium bifermentans* and C. subterminale are associated with kiwifruit vine decline, known as moria, in Italy. Plant Pathol. 69, 765–774. doi: 10.1111/ppa.13161

[B55] Testolin R. Ferguson A. R. (2009). Kiwifruit (*Actinidia* spp.) production and marketing in Italy. New Z. J. Crop Hortic. Sci. 37, 1–32. doi: 10.1080/01140670909510246

[B56] Topali C. Antonopoulou C. Chatzissavvidis C. (2024). Effect of waterlogging on growth and productivity of fruit crops. Horticulturae 10, 623. doi: 10.3390/horticulturae10060623

[B57] Vanneste J. L. (2017). The scientific, economic, and social impacts of the New Zealand outbreak of bacterial canker of kiwifruit (*Pseudomonas syringae* pv. *actinidiae*). Annu. Rev. Phytopathol. 55, 377–399. doi: 10.1146/annurev-phyto-080516-035530 28613977

[B58] Xiloyannis C. Dichio B. Mininni A. N. (2023). “Vine Nutrition and Water Requirement,” in Kiwifruit: Botany, Production and Uses. Eds. Richardson A. C. Burdon J. N. Ferguson A. R. (CAB International, Boston), 164–183.

[B59] Xu F. Cai H. Zhang X. Su M. Zhou H. Li X.. (2022). Comparison of waterlogging tolerance of three peach rootstock seedlings based on physiological, anatomical and ultra-structural changes. Horticulturae 8, 720. doi: 10.3390/horticulturae8080720

[B60] Youn-Seop J. Hye-Sung C. In-Seop L. Wol-Soo K. (2008). Assessment of waterlogging tolerance and change in net photosynthetic rate of major Actinidia species during flooding treatment. Acta Hortic. 773, 289–293. doi: 10.17660/ActaHortic.2008.773.43

[B61] Zahra N. Hafeez M. B. Shaukat K. Wahid A. Hussain S. Naseer R.. (2021). Hypoxia and Anoxia Stress: Plant responses and tolerance mechanisms. J. Agron. Crop Sci. 207, 249–284. doi: 10.1111/jac.12471

[B62] Zhang B. Sun M. Liu W. Lian M. Yang S. Peng F.. (2023). Waterlogging resistance and evaluation of physiological mechanism of three peach (Prunus persica) rootstocks. Protoplasma 260, 1375–1388. doi: 10.1007/s00709-024-01949-8 37010630

